# Efficient synthesis of π-conjugated molecules incorporating fluorinated phenylene units through palladium-catalyzed iterative C(sp^2^)–H bond arylations

**DOI:** 10.3762/bjoc.11.218

**Published:** 2015-10-28

**Authors:** Fatiha Abdelmalek, Fazia Derridj, Safia Djebbar, Jean-François Soulé, Henri Doucet

**Affiliations:** 1 Institut des Sciences Chimiques de Rennes, UMR 6226 CNRS-Université de Rennes 1 "Organométalliques, Matériaux et Catalyse", Campus de Beaulieu, 35042 Rennes, France; 2 Département de Chimie, UMMTO, University, BP 17 RP, 15000 Tizi-Ouzou, Algeria; 3 Laboratoire d’hydrométallurgie et chimie inorganique moléculaire, Faculté de Chimie, U.S.T.H.B. Bab-Ezzouar, Algeria

**Keywords:** catalysis, C–H bond arylations, desulfitative, fluorine, palladium

## Abstract

We report herein a two or three step synthesis of fluorinated π-conjugated oligomers through iterative C–H bond arylations. Palladium-catalyzed desulfitative arylation of heteroarenes allowed in a first step the synthesis of fluoroaryl-heteroarene units in high yields. Then, the next steps involve direct arylation with aryl bromides catalyzed by PdCl(C_3_H_5_)(dppb) to afford triad or tetrad heteroaromatic compounds via regioselective activation of C(sp^2^)–H bonds.

## Introduction

**Figure 1:**
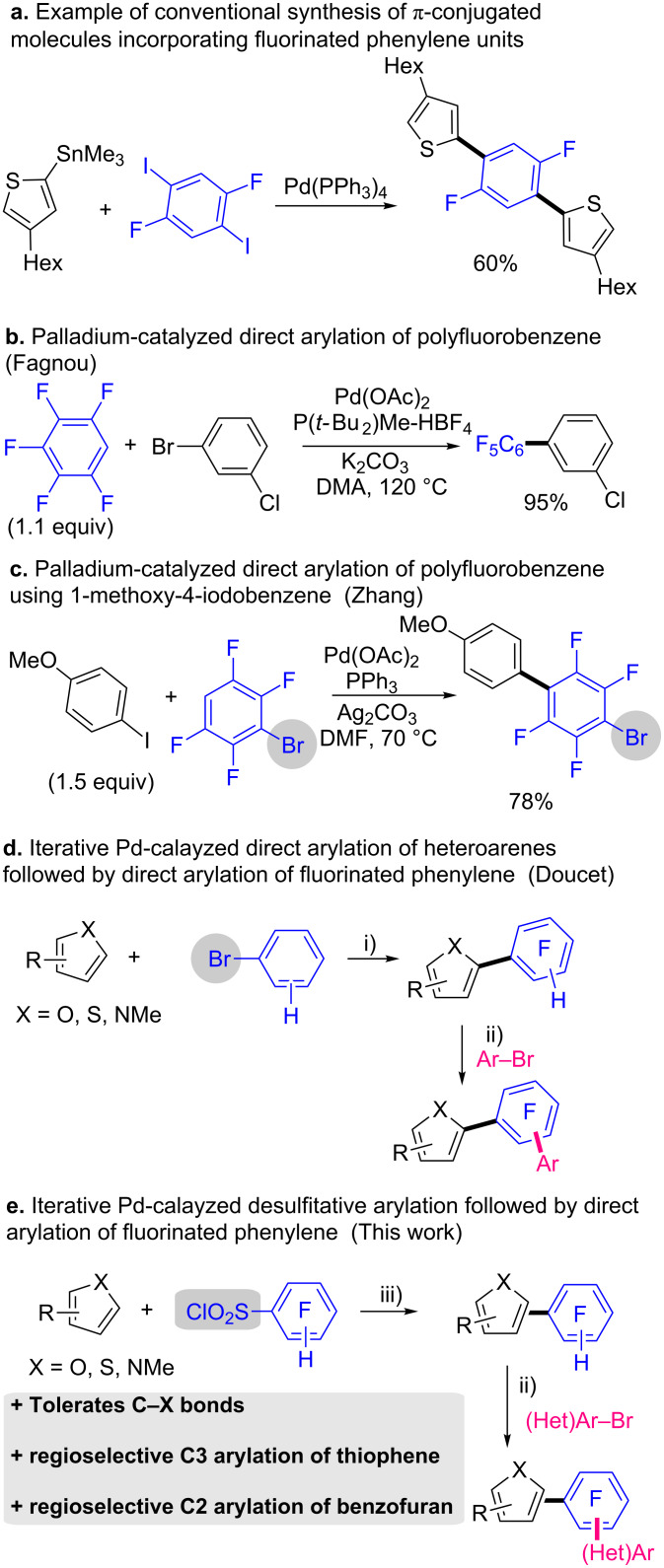
Different pathways for the synthesis of π-conjugated molecules incorporating fluorinated phenylene units. i) Pd(OAc)_2_, KOAc, DMA, 150 °C. ii) PdCl(C_3_H_5_)(dppb), KOAc, DMA, 150 °C ; ii) PdCl(C_3_H_5_)(dppb), KOAc, DMA, 150 °C; iii) PdCl_2_(CH_3_CN)_2_, Li_2_CO_3_, 1,4-dioxane, 140 °C.

Fluorinated π-conjugated oligomers increasingly receive recent interest owing to their particular applications as electronic devices (e.g., in organic solar cells) [1–4]. In addition, several fluorinated heterobiaryls such as atorvastatin, rosuvastatin, or pitavastatin have been developed as drugs and commercialized by pharmaceutical companies [5]. In both areas, fluorine atoms can modify the properties of organic compounds. It is established that fluorine atoms dramatically influence both chemical properties and reactivities owing to its electronegativity, size, lipophilicity, and electrostatic interactions. For example, introduction of fluorine into natural products can result in beneficial biological properties [6]. On the other hand, fluorinated π-conjugated oligomers have unique π-staking arrangement resulting sometimes in specific electronic properties [3]. Hence, a practical method for the synthesis of fluorinated π-conjugated oligomers that will use the specificity of fluorine atoms is highly desirable for the chemist community. Palladium-catalyzed cross-coupling reactions are one of the most powerful technologies for the efficient synthesis of π-conjugated oligomers. As example, a π-conjugated thiophene triad incorporating a difluorinated phenylene unit has been previously synthesized in 60% yield using the Stille reaction of trimethyl(thiophen-2-yl)stannane and 1,4-difluoro-2,5-diiodobenzene (Figure 1a) [3]. However, this methodology required the pre-synthesis of the stannane derivative, which is not eco-friendly. More recently, the direct C–H bond arylation has appeared as one of the most sustainable protocols for the synthesis of poly(hetero)arenes in high yields in only a few steps with the respect of the environment [7–25]. Since the reports on transition metal-catalyzed direct arylation of polyfluorobenzenes by Fagnou (Figure 1b) [26], and others [27–34], this technology has been increasingly employed for the synthesis of fluorinated π-conjugated materials, but this strategy was limited to the synthesis of end-caped fluorene oligomers [35–43]. Organic molecules containing polyfluoroaryl groups have been also synthetized through direct olefination [44]. In 2012, Zhang and co-workers reported one example of the use of 3-bromo-1,2,4,5-tetrafluorobenzene with iodoanisole as coupling partner (Figure 1c) [45]. The reaction conditions tolerate the C–Br bond on the polyfluorobenzene allowing the synthesis of (hetero)aryl triads with a fluoroarene unit. More recently, our research group also reported the synthesis of (hetero)aryl triads from fluorinated arenes bearing a C–Br bond using iterative palladium-catalyzed direct arylations (Figure 1d) [46]. In a first step, the C–Br bond of fluorinated phenylene units was involved in palladium-catalyzed direct arylations with a set of heteroarenes (e.g., thiophenes, thiazoles, furans). Then, the resulting heteroarylated polyfluorobenzenes were arylated using PdCl(C_3_H_5_)(dppb) catalyst in the presence of KOAc as the base in DMA at 150 °C using a wide range of aryl bromides as coupling partners.

On the other hand, benzenesulfonyl chlorides were recently introduced as powerful arylating agents, through metal-catalyzed C–H bond activation [47–55]. Such desulfitative direct arylations sometimes offered different regioselectivities, e.g., thiophenes were arylated in β-position instead of α-position with the classical procedure employing aryl halides as coupling partners [53,55] and benzofuran regioselectively led to C2 arylated compounds instead of the mixtures of C2 and C3 arylated products obtained with aryl bromides [56]. Moreover, these processes are very chemoselective, as the reactions only involve desulfitative coupling even in the presence of C–halogen bonds [57], which allow further orthogonal transformations. Moreover, a wide scope of benzenesulfonyl chlorides is commercially available at an affordable cost.

We report herein, a new synthetic pathway for fluorinated π-conjugated oligomers (i.e., dyads, triads, and tetrads). This novel strategy involves only palladium-catalyzed iterative C–H bond arylations. In a first step, PdCl_2_(CH_3_CN)_2_-catalyzed desulfitative regioselective arylations of heteroarenes using fluorinated benzenesulfonyl chlorides as coupling partners allowed the formation of heteroarylated polyfluorobenzenes. Then, in a second and third step, the PdCl(C_3_H_5_)(dppb)-catalyzed regioselective arylations using aryl halides as coupling partners furnished the desired triads or tetrads.

## Results and Discussion

**Scheme 1:**
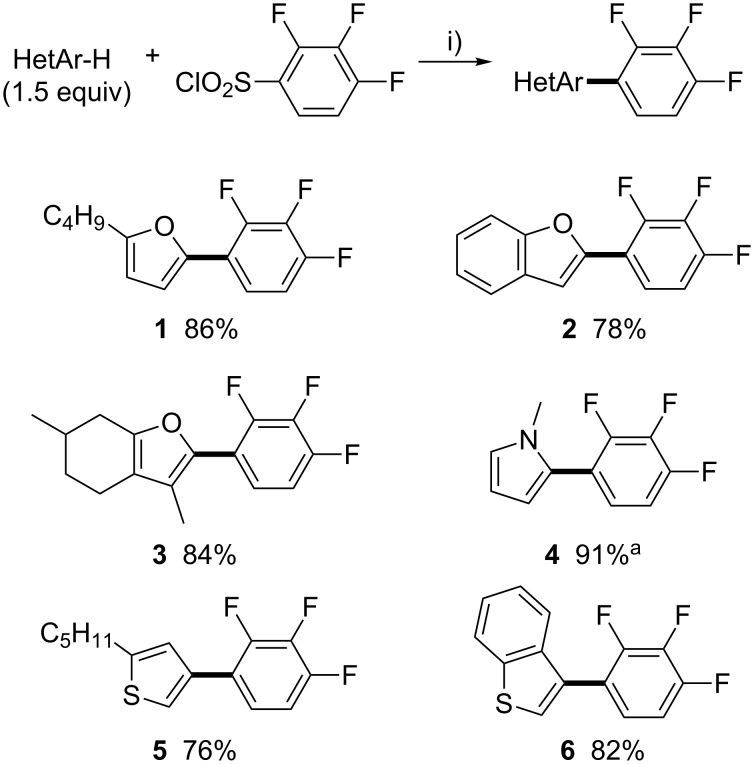
Pd-catalyzed desulfitative direct arylations of heteroarenes using 2,3,4-trifluorobenzenesulfonyl chloride as the arylating agent. i) PdCl_2_(CH_3_CN)_2_ (5 mol %), Li_2_CO_3_ (3 equiv), 1,4-dioxane, 140 °C, 48 h; ^a^The reaction was performed using 4 equiv of 1-methylpyrrole during 15 h.

We started our investigation with the synthesis of a set of heteroarenes bearing a 1,2,3-trifluorobenzene motif (Scheme 1). Using our previous reaction conditions, namely 5 mol % PdCl_2_(CH_3_CN)_2_ in the presence of 3 equiv of Li_2_CO_3_ in dioxane at 140 °C, both 2-*n*-butylfuran and benzofuran reacted with 2,3,4-trifluorobenzenesulfonyl chloride to give the C5- and C2-arylated products **1** and **2** in 86% and 78% yields. Menthofuran, in which only the C2 position is available, also smoothly reacted with 2,3,4-trifluorobenzenesulfonyl chloride to give **3** in 84% yield. Using the same reaction conditions, *N*-methylpyrrole affords the 1-methyl-2-(2,3,4-trifluorophenyl)pyrrole (**4**) in 91% yield. It is important to note that 4 equiv of *N*-methylpyrrole were used in order to prevent the formation of the 2,5-diarylated pyrrole as a side product. Finally, both 2-pentylthiophene and benzothiophene were arylated in 76% and 82% yields, respectively. Notably, the thiophene derivative was regioselectively arylated at the C4 position and the benzothiophene at the C3 position, which are challenging positions to functionalize using aryl bromides as the coupling partners [58].

**Scheme 2:**
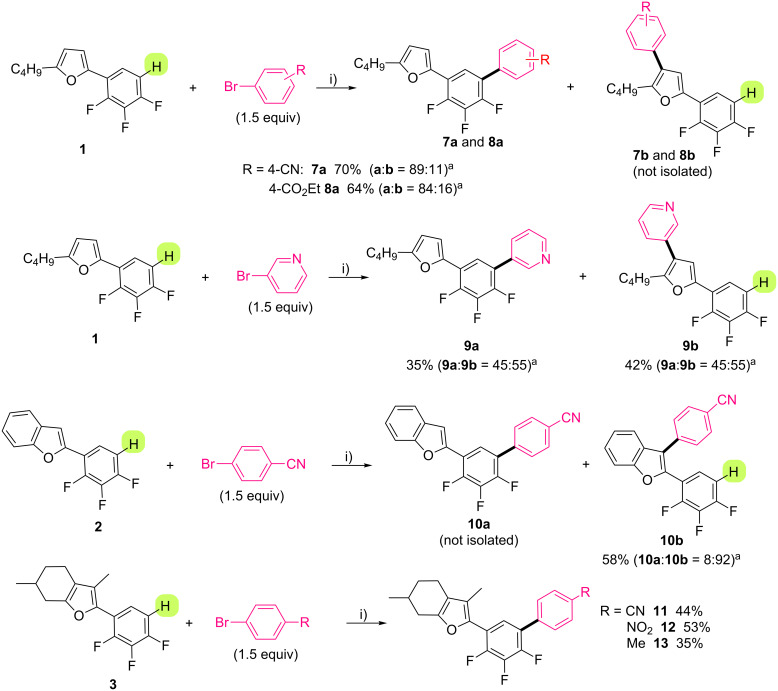
Pd-catalyzed second arylation of **1** and **2**. i) PdCl(C_3_H_5_)(dppb) (2 mol %), KOAc (2 equiv), DMA, 150 °C, 16 h. ^a^Regioselectivity (**a**:**b**) ratio determined from crude ^1^H NMR.

**Scheme 3:**
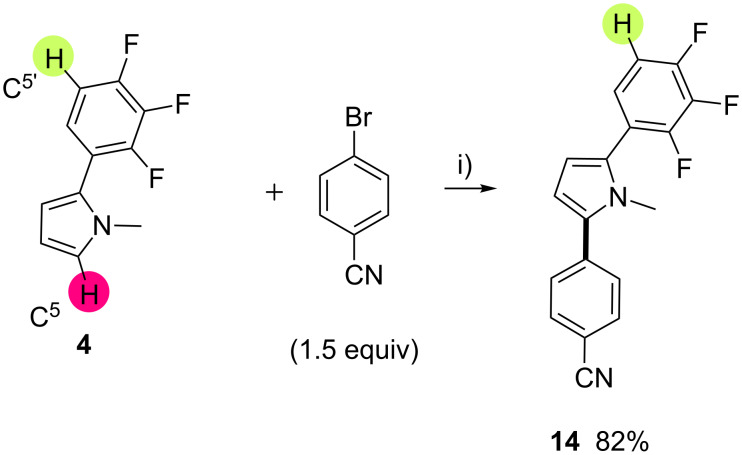
Pd-catalyzed direct regioselective arylation of 1-methyl-2-(2,3,4-trifluorophenyl)pyrrole (**4**). i) PdCl(C_3_H_5_)(dppb) (2 mol %), KOAc (2 equiv), DMA, 150 °C, 16 h.

**Scheme 4:**
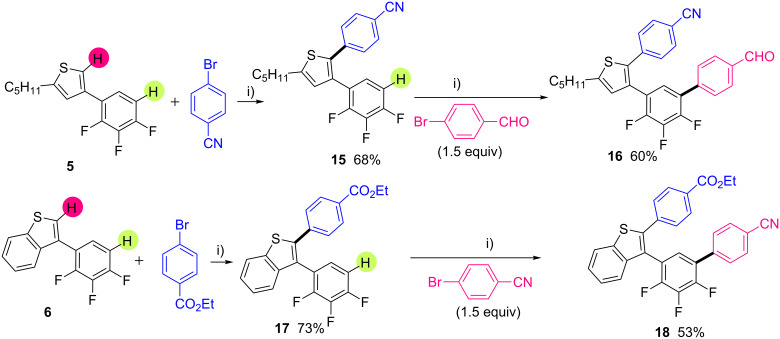
Pd-catalyzed direct regioselective arylation of 3-(2,3,4-trifluorophenyl)thiophenes. i) PdCl(C_3_H_5_)(dppb) (2 mol %), KOAc (2 equiv), DMA, 150 °C, 16 h.

Substrates **1**–**6** contain several reactive C–H bonds in palladium-catalyzed direct arylation using aryl bromides as coupling partners. Indeed, the regioselectivity of such reaction with these coupling partners needs to be investigated (Schemes 2–4). (2,3,4-Trifluorophenyl)furans **1** and **2** have two and three C–H bonds, respectively, which are susceptible to react under palladium-catalyzed direct arylation conditions, namely the C–H bond at the *ortho* position to the fluorine atom and the C–H bonds at C3 or C4 positions of the (benzo)furan (Scheme 2). We decided to employ the reaction conditions that we had previously described for both direct arylations of fluorobenzenes [46,59–60] and C3-arylation of furans [61–62] (i.e., 2 mol % PdCl(C_3_H_5_)(dppb) as catalyst associated to KOAc as base in DMA). We were pleased to find that 2-butyl-5-(2,3,4-trifluorophenyl)furan (**1**) was preferentially arylated on the electron-deficient ring whichever the aryl bromide was. For example, using 4-bromobenzonitrile or ethyl 4-bromobenzoate, the coupling products **7** and **8** were isolated in 70% and 64% yields, respectively. However, other regioisomers were detected in the crude mixtures in trace amounts (e.g., 11–16%), which resulted from the activation of the furyl C–H bond. In contrast to these examples, when the reaction was performed with a heteroaryl bromide such as 3-bromopyridine, the two regioisomers **9a** and **9b** were obtained in 45:55 ratio. On the other hand, it is well known that the C–H bond at the C-3 position of the benzofuran is very reactive in palladium-catalyzed direct arylation with aryl halides [62–64]. A similar reactivity trend was observed using 2-(2,3,4-trifluorophenyl)benzofuran (**2**) as starting material, which furnished the C3-arylated 2-(2,3,4-trifluorophenyl)benzofuran **10** in 58% yield with 92% regioselectivity. (Hetero)aryl triads containing a trifluorobenzene unit were synthetized using 2-(2,3,4-trifluorophenyl)menthofuran (**3**), in which the reactive C–H bonds are only present on the trifluorobenzene unit. Both 4-bromobenzonitrile or 4-bromonitrobenzene gave the arylated products **11** and **12** in good yields, whereas the coupling product with 4-bromotoluene, compound **13**, was isolated in 35% yield. This lower yield might be explained by a slower oxidative addition rate to the palladium with this electron-rich aryl bromide.

Next, we investigated the reactivity of 1-methyl-2-(2,3,4-trifluorophenyl)pyrrole (**4**) in a second direct arylation (Scheme 3). Using of PdCl(C_3_H_5_)(dppb) catalyst and KOAc as base in DMA, the C5 position of the pyrrole unit was arylated in 82% yield with a complete regioselectivity; whereas the C–H bond at C5’ position on the trifluorobenzene unit remained untouched. Moreover, under similar reaction conditions, using 4-bromobenzaldehyde or 4-bromopyridine as coupling partners, a C–H arylation on the trifluorobenzene moiety of **14** did not proceed.

We surveyed next the reactivity of 3-(2,3,4-trifluorophenyl)thiophene derivatives **5** and **6** in palladium catalyzed direct arylation using the same reaction conditions (Scheme 4). As expected, with 2-pentyl-4-(2,3,4-trifluorophenyl)thiophene (**5**) – which has a very reactive C–H bond at the thienyl C5-position – the arylation took place regioselectively at this position, whereas the C–H bonds on trifluorobenzene unit remained untouched. C2,C3-Diarylated thiophene **15** was obtained in 68% yield. Then, a third iterative palladium-catalyzed direct arylation was performed from **15** using the same reaction conditions and 4-bromobenzaldehyde as the coupling partner. The arylation occurred at the *ortho*-position of the fluorine atom to give **16** in 60% yield (Scheme 4, top). A similar iterative C–H bond arylation process was conducted starting from 3-(2,3,4-trifluorophenyl)benzothiophene (**6**) (Scheme 4, bottom). The first direct arylation, using ethyl 4-bromobenzoate as coupling partner, occurred at the benzothienyl C–H bond to give the C2,C3 diarylated benzothiophene **17** in a good yield of 73%. The second direct arylation allowed the formation of the tetra(hetero)aryl compound **18** in 53% yield.

**Scheme 5:**
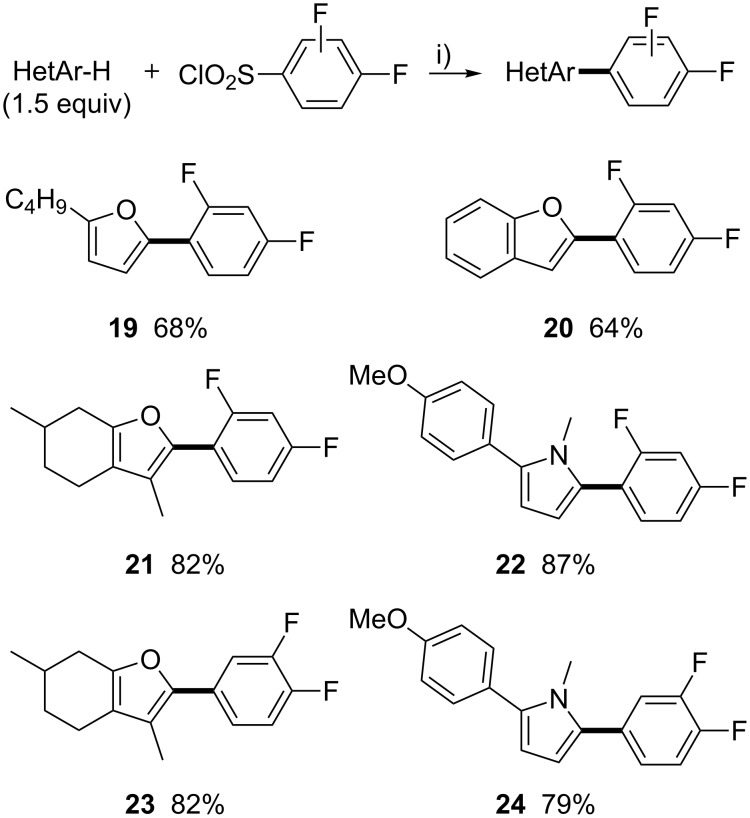
Pd-catalyzed desulfitative direct arylations of heteroarenes using difluorobenzenesulfonyl chlorides as the arylating agents. i) PdCl_2_(CH_3_CN)_2_ (5 mol %), Li_2_CO_3_ (3 equiv), 1,4-dioxane, 140 °C, 48 h.

Having demonstrated that palladium-catalyzed iterative direct arylations might be an efficient synthetic pathway for the synthesis of (hetero)aryl triads containing a trifluorobenzene unit, we decided then to turn our attention to the synthesis of (hetero)aryl triads containing a difluorobenzene unit. As previously, we synthesized a set of difluorobenzene-substituted heteroarenes using our previous palladium-catalyzed desulfitative arylation conditions (Scheme 5). 2,4-Difluorobenzenesulfonyl chloride was efficiently coupled with 2-*n*-butylfuran, benzofuran, and menthofuran to give the C2 arylated furans **19**–**21** in 64–82% yields. Next, 2-(4-methoxyphenyl)-1-methylpyrrole, in which one of the reactive C2 and C5 C–H bonds was already arylated, was subjected to the same reaction allowing the formation of C2,C5-diarylated pyrrole **22** in excellent yield. Then, 3,4-difluorobenzenesulfonyl chloride also shown an excellent reactivity as desulfitative coupling partner with menthofuran and 2-(4-methoxyphenyl)-1-methylpyrrole to furnish the arylated heteroarenes **23** and **24** in 82% and 79% yields, respectively.

**Scheme 6:**
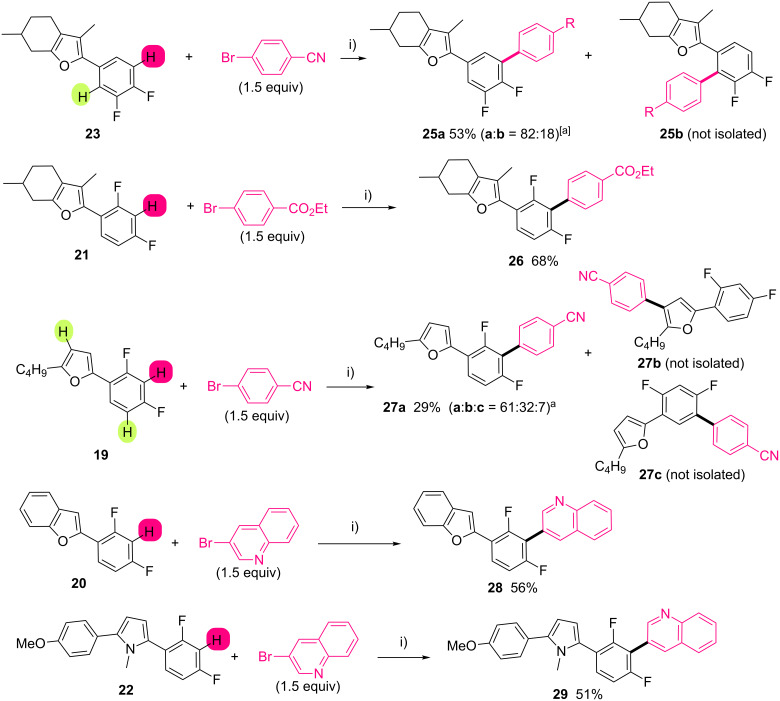
Pd-catalyzed second direct regioselective arylation of difluorophenylheteroarenes **19**-**23**. i) PdCl(C_3_H_5_)(dppb) (2 mol %), KOAc (2 equiv), DMA, 150 °C, 16 h. [a] C5/C2 regioisomers ratio determined on crude ^1^H NMR ; [b] C3'/C3/C5' regioisomers ratio determined on crude ^1^H NMR.

With C2-(difluorophenyl)heteroarenes **19**–**24** in hands, we studied their reactivity in Pd-catalyzed direct arylations using PdCl(C_3_H_5_)(dppb) as catalyst in the presence of KOAc as the base and aryl bromide as coupling partners (Scheme 6). On 2-(3,4-difluorophenyl)menthofuran (**23**), the two C–H bonds at *ortho*-position to a fluorine atom might be arylated under this reaction conditions. Using 4-bromobenzaldehyde as coupling partner, a mixture of two regioisomers in 82:18 ratio was obtained, and the C5 arylation product **25** (major regioisomer) was isolated after flash chromatography in 53% yield as pure product. It is important to note that **25** results from the activation of the less hindered C–H bond. When the reaction was performed with 2-(2,4-difluorophenyl)menthofuran (**21**), the position flanked by two fluorine atoms was the most reactive. Only the regioisomer **26** was observed in crude GC–MS and ^1^H NMR and was isolated in 68% yield. Using the more challenging substrate 2-butyl-5-(2,4-difluorophenyl)furan (**19**) with 4-bromobenzonitrile, a mixture of three regioisomers was formed in a 61:32:7 ratio. Only the major regioisomer **27**, in which the arylation happend at the C–H bond flanked by the two fluorine atoms, has been isolated in pure form. The other regioisomers might result from the activation of a C–H bond on the furan ring and of the other C–H bond at *ortho*-position to the fluorine atom. In contrast to (2,3,4-trifluorophenyl)benzofuran **2** – in which the benzofuryl C–H bond is the most reactive – 2-(2,4-difluorophenyl)benzofuran (**20**) only reacted through the activation of the electron-deficient phenyl ring to afford the heteroaryl triad **28** in 56% yield. We imputed this shift of regioselectivity to the high acidity of the C–H bond flanked by the two fluorine atoms. Then, the regioselectivity of Pd-catalyzed second direct arylation was investigated with 2-(2,4-difluorophenyl)-5-(4-methoxyphenyl)-1-methylpyrrole (**22**) and 2-(3,4-difluorophenyl)-5-(4-methoxyphenyl)-1-methylpyrrole (**24**). As expected, in the presence of the 2,4-difluorophenyl motif, the arylation exclusively took place between the two fluorine atoms to allow the formation of tetramer **29** in 51% yield.

**Scheme 7:**
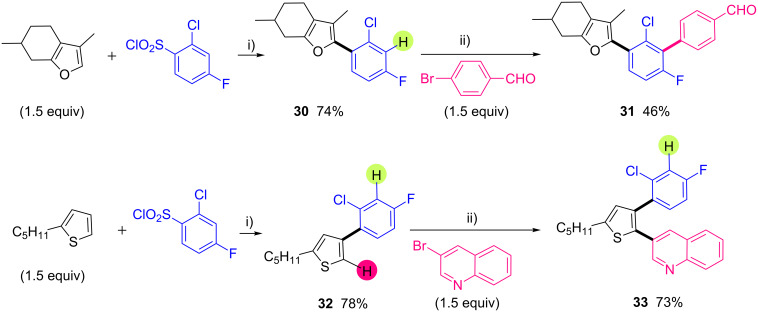
Pd-catalyzed iterative direct arylations of heteroarenes–fluorobenzene triads and tetrad. i) PdCl_2_(CH_3_CN)_2_ (5 mol %), Li_2_CO_3_ (3 equiv), 1,4-dioxane, 140 °C, 48 h; ii) PdCl(C_3_H_5_)(dppb) (2 mol %), KOAc (2 equiv), DMA, 150 °C, 16 h.

We also investigated iterative direct arylations for the construction of (hetero)aryl triads or tetrads containing a mono-fluorinated phenyl motif (Scheme 7). In 2006, it has been already demonstrated that the palladium-catalyzed direct arylation of fluorobenzene proceeds in very low yield [26]. Based on our previous works, in which we have demonstrated that a halogen atom such as chlorine or bromine at the C3 or C4 position enhances its reactivity [65], we decided to use 2-chloro-4-fluorobenzenesulfonyl chloride as the first arylating agent. We firstly selected menthofuran as starting material for the synthesis of (hetero)aryl triads (Scheme 7, top). Using our conditions for desulfitative arylation, 2-(2-chloro-4-fluorophenyl)menthofuran (**30**) was obtained in 74% yield. Then, a second arylation was performed using 2 mol % of PdCl(C_3_H_5_)(dppb) catalyst and KOAc in DMA. We were pleased to find that only one regioisomer was formed, as the arylation exclusively occurred at the C–H bond between fluorine and chlorine atoms. The desired (hetero)aryl triad **31** was isolated in moderate yield, due to the formation of a relatively large amount of homocoupling product of the aryl bromide partner. On the other hand, we also performed similar sequential C–H bond arylations from 2-pentylthiophene as starting material (Scheme 7, bottom). The desulfitative arylation occurred at the expected the C4-position to afford 4-(2-chloro-4-fluorophenyl)-2-pentylthiophene (**32**) in 78% yield. Then, we found that the thienyl C5–H bond reacted faster than the phenyl C–H bond at the *ortho*-position to chloro and fluorine atoms. Indeed using 3-bromoquinoline, the (hetero)aryl triad **33** was obtained in 73% yield as a single regioisomer.

**Scheme 8:**
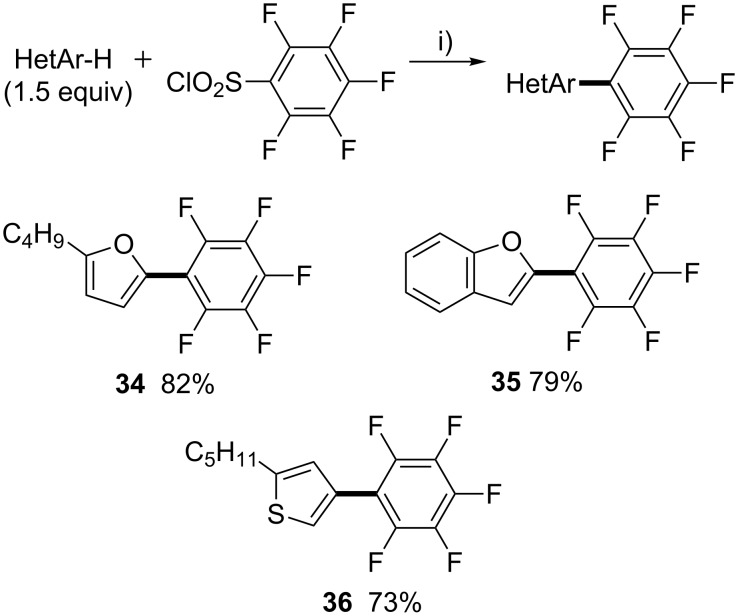
Reactivity of pentafluorobenzenesulfonyl chloride in Pd-catalyzed direct desulfitative arylation of heteroarenes. i) PdCl_2_(CH_3_CN)_2_ (5 mol %), Li_2_CO_3_ (3 equiv), 1,4-dioxane, 140 °C, 48 h.

On the other hand, Zhang and co-workers developed a straightforward and practical method for the synthesis of end-capped pentafluorobenzene-substituted heteroarenes, which are an important class of materials, through palladium-catalyzed oxidative cross-coupling of perfluoroarenes with aromatic heterocycles [36]. Therefore, we decided to investigate the reactivity of pentafluorobenzenesulfonyl chloride for the construction of end-capped pentafluorobenzene-substituted heteroarenes through direct desulfitative arylation (Scheme 8). We were pleased to find that using standard reaction conditions, 2-*n*-butylfuran and benzofuran were arylated at C5- or C2-positions in high yields. Moreover, this methodology allowed the synthesis of challenging C4-arylated thiophene **36** in 73% yield.

## Conclusion

In summary, we have developed an efficient synthesis of complex heteroaryl triads and tetrads through palladium-catalyzed iterative direct arylations. Tri-, di-, and monofluorophenyl-substituted heteroarenes were synthesized in high yields and high regioselectivities through a desulfitative direct arylation of several heteroarenes using fluorinated arylsulfonyl chlorides. Then, direct arylation of the (poly)fluorophenyl units was performed using aryl bromides as coupling partners. In some specific cases, we have demonstrated that C2−H bonds of (benzo)thiophene, C5−H bonds of pyrrole, and C3−H bonds of (benzo)furans are more reactive than the C−H bonds of tri- and fluorobenzenes toward palladium-catalyzed direct arylation. This strategy allows the straightforward synthesis of heteroarylated polyfluorobiphenyls in good yields via two or three sequential iterative palladium-catalyzed direct arylations.

## Supporting Information

File 1 Experimental and analytical data.
