# Supplementary material for: Efficient synthesis of π-conjugated molecules incorporating fluorinated phenylene units through palladium-catalyzed iterative C(sp2)–H bond arylations
Source: Beilstein J Org Chem. 2015 Oct 28;11:2012–20. doi: 10.3762/bjoc.11.218 (PMC4661000; doi:10.3762/bjoc.11.218)
Supplement: File 1 — Experimental and analytical data. [file Beilstein_J_Org_Chem-11-2012-s001.pdf]

Supporting Information

for

Efficient synthesis of π-conjugated molecules incorporating fluorinated phenylene units through palladium-catalyzed iterative C(sp^2)–H bond arylations

Fatiha Abdelmalek[1,2], Fazia Derridj[1,2,3], Safia Djebbar[3], Jean-François Soulé*[1] and Henri Doucet*[1]

Address: [1]Institut des Sciences Chimiques de Rennes, UMR 6226 CNRS-Université de Rennes 1 "Organométalliques, Matériaux et Catalyse", Campus de Beaulieu, 35042 Rennes, France, [2]Département de chimie, UMMTO, University, BP 17 RP, 15000 Tizi-Ouzou, Algeria and [3] Laboratoire d'hydrométallurgie et chimie inorganique moléculaire, Faculté de Chimie, U.S.T.H.B. Bab-Ezzouar, Algeria

Email: Jean-François Soulé - jean-francois.soule@univ-rennes1.fr; Henri Doucet - henri.doucet@univ-rennes1.fr

*Corresponding author

Experimental and analytical data

All reactions were carried out under argon atmosphere using standard Schlenk techniques. 1,4-Dioxane and DMA were purchased from Acros Organics and were not purified before use. ^{1}H NMR spectra were recorded on a Bruker GPX (400 MHz) spectrometer. Chemical shifts (δ) were reported in parts per million relative to residual chloroform (7.26 ppm for ^{1}H; 77.0 ppm for ^{13}C), coupling constants (*J*) were reported in Hertz. ^{1}H NMR assignment abbreviations were the following: singlet (s), doublet (d), triplet (t), quartet (q), doublet of doublets (dd), doublet of triplets (dt), and multiplet (m). ^{13}C NMR spectra were recorded at 100 MHz on the same spectrometer and reported in ppm. Direct analysis in real time (DART) mass spectra were recorded on a JEOL JMS-T100TD mass spectrometer. All reagents were weighed and handled in air.

Preparation of the PdCl(dppb)(C_3H_5) catalyst: According the procedure described in [1], An oven-dried 40 mL Schlenk tube equipped with a magnetic stirring bar under argon atmosphere, was charged with $[Pd(C_3H_5)Cl]_2$ (182 mg, 0.5 mmol) and dppb (426 mg, 1 mmol). 10 mL of anhydrous dichloromethane were added, and then the solution was stirred at room temperature for 20 minutes. The solvent was removed in vacuum. The yellow powder obtained was used without purification. ^{31}P NMR (81 MHz, $CDCl_3$) δ (ppm) = 19.3 (s).

Procedure A (desulfitative arylation): In a similar manner as described in [2], to a 5 mL oven-dried Schlenk tube, arylsulfonyl chloride (2.5 or 1 mmol), heteroarenes derivatives (3.75 or 1.5 mmol), Li_2CO_3

(0.55 g or 0.22 g, 7.5 mmol or 3 mmol), 1,4-dioxane (5 mL) and bis(acetonitrile)dichloropalladium(II) (32.3 or 12 mg, 0.125 or 0.05 mmol) were successively added. The reaction mixture was evacuated by vacuum-argon cycles (5 times) and stirred at 140 °C (oil bath temperature) for 16–48 hours (see tables and schemes). After cooling the reaction mixture to room temperature and concentration, the crude mixture was purified by silica column chromatography to afford the desired arylated products.

Procedure B (direct arylation with aryl bromides): In a similar manner as described in [3], to a 5 mL oven dried Schlenk tube, fluorinated heteroaryl (0.5 mmol), aryl bromide (0.75 mmol, 1.5 equiv.), AcOK (100 mg, 1 mmol), DMA (2 mL) and $PdCl(C_3H_5)$(dppb) (6 mg, 0.01 mmol, 2 mol%) were successively added. The reaction mixture was evacuated by vacuum-argon cycles (5 times) and stirred at 150 °C (oil bath temperature) for 16-48 hours (see tables and schemes). After cooling the reaction at room temperature and concentration, the crude mixture was purified by silica column chromatography to afford the desired arylated products.

2-*n*-Butyl-5-(2,3,4-trifluorophenyl)furan (1): Following the procedure **A** using 2-*n*-butylfuran (525 µl, 3.75 mmol) and 2,3,4-trifluorobenzenesulfonyl chloride (352 µL, 2.5 mmol). The residue was purified by flash chromatography on silica gel (pentane, 100) to afford the desired compound **1** (0.547 g, 86%) as a pale yellow oil.

^{1}H NMR (400 MHz, $CDCl_3$) δ (ppm) 7.47 (ddd, *J* = 5.7, 7.8 and 9.1 Hz, 1H), 6.98 (ddt, *J* = 2.1, 7.1 and 9.1 Hz, 1H), 6.71 (t, *J* = 3.6 Hz, 1H), 6.11 (d, *J* = 3.3 Hz, 1H), 2.69 (t, *J* = 7.6 Hz, 2H), 1.68 (quint , *J* = 7.6 Hz, 2H), 1.42 (sext , *J* = 7.6 Hz, 2H), 0.96 (t, *J* = 7.6 Hz, 3H).

^{13}C NMR (100 MHz, $CDCl_3$) δ (ppm) 157.0, 149.6 (dd, *J* = 11.2 Hz and 247.1), 147.2 (dd, *J* = 14.0 Hz and 255.5), 144.3, 140.4 (td, *J* = 16.1 Hz and 247.8), 118.6, 117.3 (dd, *J* = 3.7 and 9.5 Hz), 111.9 (d, *J* = 17.4 Hz), 111.0 (d, *J* = 11.4 Hz), 107.2, 30.1, 27.7, 22.3, 13.6.

Elemental analysis: calcd (%) for $C_{14}H_{13}F_3O$ (254.25): C 66.14, H 5.15; found: C 66.37, H 5.29.

2-(2,3,4-Trifluorophenyl)benzofuran (2): Following the procedure **A** using benzofuran (413 µl, 3.75 mmol) and 2,3,4-trifluorobenzenesulfonyl chloride (352 µL, 2.5 mmol). The residue was purified by flash chromatography on silica gel (pentane, 100%) to afford the desired compound **2** (0.483 g, 78%) as a white solid (mp = 120–124 ºC).

^{1}H NMR (400 MHz, $CDCl_3$**)** δ (ppm) 7.70 (ddd, *J* = 5.8, 7.8 and 9.1 Hz, 1H), 7.63 (d, *J* = 7.8 Hz, 1H), 7.53 (d, *J* = 8.1 Hz, 1H), 7.36 (t, *J* = 7.8 Hz, 1H), 7.29 (t, *J* = 7.8 Hz, 1H), 7.19 (d, *J* = 3.7 Hz, 1H), 7.05 (ddd, *J* = 2.1, 6.9 and 9.2 Hz, 1H).

^{13}C NMR (100 MHz, $CDCl_3$) δ (ppm) 154.1, 150.7 (dd, *J* = 13.1 and 251.3 Hz), 148.5 (dd, *J* = 13.1 and 254.0 Hz), 147.8, 140.3 (td, *J* = 15.8 and 249.3 Hz), 128.9, 125.1, 123.2, 121.4, 120.3 (ddd, *J* = 3.4, 4.3 and 8.0 Hz), 116.5 (dd, *J* = 4.3, and 8.9 Hz), 112.3 (dd, *J* = 3.9 and 17.7 Hz), 111.0, 106.6 (d, *J* = 12.2 Hz).

HRMS (DART) m/z: [M + H]+ Calcd for $C_{14}H_8F_3O$ 249.0527; Found 249.0496.

3,6-Dimethyl-2-(2,3,4-trifluorophenyl)-4,5,6,7-tetrahydrobenzofuran (3): Following the procedure **A** using menthofuran (387 µl, 3.75 mmol) and 2,3,4-trifluorobenzenesulfonyl chloride (352 µL, 2.5 mmol), the residue was purified by flash chromatography on silica gel (pentane, 100%) to afford the desired compound **3** (0.588 g, 84%) as a white solid (mp = 79–84 ºC).

^{1}H NMR (400 MHz, $CDCl_3$) δ (ppm) 7.20 (dtd, *J* = 2.6 and 6.8 and 9.0 Hz, 1H), 6.99 (ddt, *J* = 2.2, 7.2 and 9.3 Hz, 1H), 2.73 (dd, *J* = 5.4 and 16.4 Hz, 1H), 2.49-2.32 (m, 2H), 2.24 (dd, *J* = 9.5 and 16.5 Hz, 1H), 2.0 (d, *J* = 3.1 Hz, 3H), 1.99-1.92 (m, 1H), 1.91-1.84 (m, 1H), 1.46-1.35 (m, 1H), 1.12 (d, *J* = 6.7 Hz, 3H).

^{13}C NMR (100 MHz, $CDCl_3$) δ (ppm) 151.3, 150.2 (dd, *J* = 10.8 and 240.3 Hz), 147.7 (dd, *J* = 13.0 and 240.3 Hz), 140.4 (td, *J* = 15.4 and 251.1 Hz), 140.2, 122.9 (td, *J* = 3.9 and 7.7 Hz), 119.6, 119.3, 118.1 (dd, *J* = 3.5 and 12.2 Hz), 112.0 (dd, *J* = 3.9 and 17.4 Hz), 31.4, 31.2, 29.6, 21.5, 20.1, 9.1 (d, *J* = 6.7 Hz).

Elemental analysis: calcd (%) for $C_{16}H_{15}F_3O$ (280.29): C 68.56, H 5.39; found: C 68.45, H 5.66.

1-Methyl-2-(2,3,4-trifluorophenyl)pyrrole (4): Following the procedure **A** using 1-methylpyrrole (888 µl, 10 mmol) and 2,3,4-trifluorobenzenesulfonyl chloride (352 µL, 2.5 mmol). The residue was purified by flash chromatography on silica gel (pentane, 100%) to afford the desired compound **4** (0.480 g, 91%) as a colorless oil.

^{1}H NMR (400 MHz, $CDCl_3$) δ (ppm) 7.13-6.99 (m, 2H), 6.81 (t, *J* = 2.3 Hz, 1H), 6.27 (d, *J* = 2.3 Hz, 2H), 3.60 (s, 3H).

^{13}C NMR (100 MHz, $CDCl_3$) δ (ppm) 150.6 (dd, *J* = 11.8 and 248.7 Hz), 148.9 (dd, *J* = 11.8 and 246.4 Hz), 140.2 (td, *J* = 14.9 and 250.4 Hz), 126.0, 125.3 (td, *J* = 2.8 and 7.4 Hz), 124.1, 119.0 (dd, *J* = 3.9 and 12.7 Hz), 111.9 (dd, *J* = 3.5 and 16.9 Hz), 110.7, 108.1, 34.5 (d, *J* = 4.5 Hz).

Elemental analysis: calcd (%) for $C_{11}H_8F_3N$ (211.19): C 62.56, H 3.82; found: C 62.89, H 4.01.

2-Pentyl-4-(2,3,4-trifluorophenyl)thiophene (5): Following the procedure **A** using 2-pentylthiophene (605 µl, 3.75 mmol) and 2,3,4-trifluorobenzenesulfonyl chloride (352 µL, 2.5 mmol). The residue was purified by flash chromatography on silica gel (pentane, 100%) to afford the desired compound **5** (0.540 g, 76%) as a colorless oil.

^{1}H NMR (400 MHz, $CDCl_3$) δ (ppm) 7.38 (s, 1H), 7.30-7.23 (m, 1H), 7.08 (s, 1H), 7.01 (ddt, *J* = 2.2, 7.1 and 9.2 Hz, 1H), 2.89 (t, *J* = 7.7 Hz, 2H), 1.77 (quint , *J* = 7.7 Hz, 2H), 1.46-1.39 (m, 4H), 0.98 (t, *J* = 7.7 Hz, 3H).

^{13}C NMR (100 MHz, $CDCl_3$) δ (ppm) 149.9 (dd, *J* = 13.5 and 249.5 Hz), 148.7 (dd, *J* = 13.5 and 254.5 Hz), 146.5, 140.5 (td, *J* = 17.0 and 250.1 Hz), 133.3, 124.0 (d, *J* = 3.1 Hz), 122.4 (td, *J* = 4.1 and 7.7 Hz), 121.8 (dd, *J* = 3.8 and 10.1 Hz), 121.4 (d, *J* = 6.8 Hz), 111.9 (dd, *J* = 4.1 and 17.2 Hz), 31.3, 30.0, 22.4, 13.9.

Elemental analysis: calcd (%) for $C_{15}H_{15}F_3S$ (284.34): C 63.36, H 5.32; found: C 63.68, H 5.71.

3-(2,3,4-Trifluorophenyl)benzothiophene (6): Following the procedure **A** using benzothiophene (438 µl, 3.75 mmol) and 2,3,4-trifluorobenzenesulfonyl chloride (352 µL, 2.5 mmol). The residue was purified by flash chromatography on silica gel (pentane, 100%) to afford the desired compound **6** (0.542 g, 82%) as a white solid (mp = 118–123 ºC).

^{1}H NMR (400 MHz, $CDCl_3$) δ (ppm) 7.93 (dd, *J* = 3.2 and 6.1 Hz, 1H), 7.69-7.64(m, 1H), 7.50 (s, 1H), 7.41 (dd, *J* = 3.2 and 6.1 Hz, 2H), 7.25-7.20 (m, 1H), 7.13-7.05 (m, 1H).

^{13}C NMR (100 MHz, $CDCl_3$) δ (ppm) 150.7 (ddd, *J* = 2.3, 9.1 and 249.7 Hz), 149.1 (ddd, *J* = 2.3, 9.1 and 249.7 Hz), 140.4 (td, *J* = 16.1 and 252.7 Hz), 140.0, 137.6, 129.2, 126.2, 124.7, 124.5, 123.7 (d, *J* = 8.8 Hz), 122.8, 122.5, 121.0 (dd, *J* = 3.8 and 12.6 Hz), 112.1 (dd, *J* = 4.2 and 17.1 Hz).

Elemental analysis: calcd (%) for $C_{14}H_7F_3S$ (264.27): C 63.63, H 2.67; found: C 63.89, H 2.94.

5'-(5-*n*-Butylfuran-2-yl)-2',3',4'-trifluoro-[1,1'-biphenyl]-4-carbonitrile (7): Following the procedure **B** using 2-butyl-5-(2,3,4-trifluorophenyl)furan (**1**) (127 mg, 0.5 mmol) and 4-bromobenzene (137 mg, 0.75 mmol). The residue was purified by flash chromatography on silica gel (pentane–Et_2O, 95:5) to afford the desired compound **7** (0.124 g, 70%) as a orange solid (mp = 82–88 ºC).

^{1}H NMR (400 MHz, $CDCl_3$) δ (ppm) 7.78 (d, *J* = 8.2 Hz, 2H), 7.67 (d, *J* = 8.2 Hz, 2H), 7.57 (ddd, *J* = 2.3, 7.5 and 8.2 Hz, 1H), 6.79 (t, *J* = 3.6 Hz, 1H), 6.15 (d, *J* = 3.3 Hz, 1H), 2.69 (t, *J* = 7.8 Hz, 2H), 1.67 (quint, *J* = 7.8 Hz, 2H), 1.46-1.35 (m, 2H), 0.95 (t, *J* = 7.4 Hz, 3H).

^{13}C NMR (100 MHz, $CDCl_3$) δ (ppm) 157.5, 148.8 (td, J = 17.7 and 252.3 Hz), 143.7, 140.6 (dd, *J* = 17.3 and 261.5 Hz), 138.7, 132.4, 129.6, 124.4 (dd, *J* = 3.6 and 9.9 Hz), 119.0, 118.5, 117.4 (dd, *J* = 4.1 and 9.3 Hz), 112.1, 112.0, 111.9, 107.6, 30.2, 27.7, 22.3, 13.8.

Elemental analysis: calcd (%) for $C_{21}H_{16}F_3NO$ (355.36): C 70.98, H 4.54; found: C 71.25, H 4.38.

Ethyl 5'-(5-*n*-butylfuran-2-yl)-2',3',4'-trifluoro-[1,1'-biphenyl]-4-carboxylate (8): Following the procedure **B** using 2-butyl-5-(2,3,4-trifluorophenyl)furan (**1**) (127 mg, 0.5 mmol) and ethyl 4-bromobenzoate (172 mg, 0.75 mmol) The residue was purified by flash chromatography on silica gel (pentane-Et_2O, 85:15) to afford the desired compound **8** (0.129 g, 64%) as a colorless oil.

^{1}H NMR (400 MHz, $CDCl_3$) δ (ppm) 8.15 (d, *J* = 8.3 Hz, 2H), 7.63 (d, *J* = 8.3 Hz, 2H), 7.59 (dt, J = 2.2 and 7.7 Hz, 1H), 6.78 (t, *J* = 3.6 Hz, 1H), 6.14 (d, *J* = 3.3 Hz, 1H), 4.42 (q, *J* = 7.1 Hz, 2H), 2.69 (t, *J* = 7.1 Hz, 2H), 1.71-1.57 (m, 4H), 1.42 (t, *J* = 6.7 Hz, 3H), 0.95 (t, *J* = 7.4 Hz, 3H).

^{13}C NMR (100 MHz, $CDCl_3$) δ (ppm) 166.2, 157.3, 146.8 (dd, *J* = 19.3 and 150.2 Hz), 146.5 (dd, *J* = 19.3 and 150.2 Hz), 144.1, 143.0 (dd, *J* = 12.0 and 241.3 Hz), 138.6, 130.3, 129.9, 128.9, 125.4 (dd, *J* = 3.8 and 12.0 Hz), 119.3, 117.1 (dd, *J* = 3.4 and 9.1 Hz), 111.7 (d, *J* = 11.3 Hz), 107.5, 61.2, 30.1, 27.8, 22.3, 14.4, 14.8.

Elemental analysis: calcd (%) for $C_{23}H_{21}F_3O_3$ (402.41): C 68.65, H 5.26; found: C 68.94, H 5.49.

3-(5-(5-*n*-Butylfuran-2-yl)-2,3,4-trifluorophenyl)pyridine (9a) and 3-(2-butyl-5-(2,3,4-trifluorophenyl)furan-3-yl)pyridine (9b): Following the procedure **B** using 2-butyl-5-(2,3,4-trifluorophenyl)furan (**1**) (127 mg, 0.5 mmol) and 3-bromopyridine (119 mg, 0.75 mmol). The residue was purified by flash chromatography on silica gel (pentane–Et_2O, 70:30) to afford the desired compound **9a** (0.058 g, 35%) and **9b** (0.070 g, 42%) as colorless oils.

9a ^{1}H NMR (400 MHz, $CDCl_3$) δ (ppm) 8.88 (brs, 1H), 8.72 (brs, 1H), 7.98 (d, *J* = 7.0 Hz, 1H), 7.60 (t, *J* = 7.1 Hz, 1H), 7.55-7.47 (m, 1H), 6.79 (t, *J* = 3.7 Hz, 1H), 6.15 (d, *J* = 3.2 Hz, 1H), 2.70 (t, *J* = 7.43 Hz, 2H), 1.67 (quint, *J* = 7.3 Hz, 2H), 1.41 (sext, *J* = 7.2 Hz, 2H), 0.95 (t, *J* = 7.3 Hz, 3H).

9a ^{13}C NMR (100 MHz, $CDCl_3$) δ (ppm) 157.5, 149.4, 147.0 (dd, *J* = 13.6 and 255.4 Hz), 144.4 (td, *J* = 13.6 and 255.4 Hz), 143.9 (m), 139.2 (dd, *J* = 13.6 and 255.4 Hz), 136.5, 130.4 (m), 126.6 (m), 119.1, 117.4 (m), 114.7 (m), 111.9 (d, *J* = 11.2 Hz), 107.6, 30.2, 27.8, 22.3, 13.8.

9b ^{1}H NMR (400 MHz, $CDCl_3$) δ (ppm) 8.72 (brs, 1H), 8.58 (brs, 1H), 7.76 (d, *J* = 7.7 Hz, 1H), 7.56-7.49 (m, 1H), 7.39 (brs, 1H), 7.03 (q, *J* = 8.2 Hz, 1H), 6.92 (d, *J* = 3.4 Hz, 1H), 2.84 (t, *J* = 7.2 Hz, 2H), 1.74 (quint, *J* = 7.2 Hz, 2H), 1.41 (sext, *J* = 7.2 Hz, 2H), 0.93 (t, *J* = 7.3 Hz, 3H).

9b ^{13}C NMR (100 MHz, $CDCl_3$) δ (ppm) 153.2, 150.5 (dd, *J* = 13.6 and 255.4 Hz), 148.2 (m), 147.6 (dd, *J* = 13.6 and 255.4 Hz), 147.5, 144.7, 140.4 (td, *J* = 13.6 and 246.9 Hz), 135.4, 130.0, 123.8, 119.9, 119.0 (td, *J* = 4.3 and 7.4 Hz), 116.7 (dd, *J* = 2.9 and 9.4 Hz), 112.4 (dd, *J* = 3.2 and 16.7 Hz), 111.2 (d, *J* = 10.1 Hz), 30.7, 26.7, 22.5, 13.8.

Elemental analysis: calcd (%) for $C_{19}H_{16}F_3NO$ (331.33): C 68.87, H 4.87; found: C 68.61, H 5.04.

4-(2-(2,3,4-Trifluorophenyl)benzofuran-3-yl)benzonitrile (10): Following the procedure **B** using 2-(2,3,4-trifluorophenyl)benzofuran **2** (248 mg, 1 mmol) and 4-bromobenzonitrile (273 mg, 1.5 mmol). The residue was purified by flash chromatography on silica gel (pentane–Et_2O, 75:25) to afford the desired compound **10** (0.203 g, 58%) as a pale yellow solid (mp = 132–136 ºC).

^{1}H NMR (400 MHz, $CDCl_3$) δ (ppm) 7.72 (d, *J* = 8.2 Hz, 2H), 7.61 (dd, *J* = 7.7 and 9.7 Hz, 2H) 7.53 (d, *J* = 8.2 Hz, 2H), 7.43 (dd, *J* = 7.33, 8.17 Hz, 1H), 7.37-7.29 (m, 2H), 7.06 (ddt, *J* = 2.1, 6.9, and 9.1 Hz, 1H).

^{13}C NMR (100 MHz, $CDCl_3$) δ (ppm) 154.8 (t, *J* = 19.9 Hz), 145.0, 137.3 (t, *J* = 8.3 Hz), 133.6 (d, *J* = 6.5 Hz), 129.0 (ddd, *J* = 4.5, 8.2 and 237.9 Hz), 128.5 (d, *J* = 6.4 Hz), 126.7 (d, *J* = 7.9 Hz), 126.6 (ddd, *J* = 4.5, 8.2

and 237.9 Hz), 125.1 (d, J = 8.8 Hz), 124.5 (d, 117.4 (td, J = 5.2 and 252.3 Hz), 123.8 (m), 123.0 (d, J = 7.4 Hz), 120.7 (d, J = 9.2 Hz), 119.1 (d, J = 8.4 Hz), 113.7 (dd, J = 3.9 and 17.8 Hz), 112.6 (d, J = 7.8 Hz), 112.0 (dd, J = 4.5 and 18.1 Hz), 111.5 (t, J = 9.0 Hz), 110.9 (d, J = 9.5 Hz).

Elemental analysis: calcd (%) for $C_{21}H_{10}F_3NO$ (349.30): C 72.21, H 2.89; found: C 72.56, H 3.17.

5'-(3,6-Dimethyl-4,5,6,7-tetrahydrobenzofuran-2-yl)-2',3',4'-trifluoro-[1,1'-biphenyl]-4-carbonitrile (11): Following the procedure **B** using 3,6-dimethyl-2-(2,3,4-trifluorophenyl)-4,5,6,7-tetrahydrobenzofuran (**3**) (280 mg, 1 mmol) and 4-bromobenzonitrile (273 mg, 1.5 mmol), the residue was purified by flash chromatography on silica gel (pentane–Et_2O, 75:25) to afford the desired compound **11** (0.168 g, 44%) as a yellow solid (mp = 92–95 ºC).

^{1}H NMR (400 MHz, $CDCl_3$) δ (ppm) 7.76 (d, J = 8.2 Hz, 2H), 7.65 (d, J = 8.2 Hz, 2H), 7.32 (dt, J = 2.2 and 7.5 Hz, 1H), 2.72 (dd, J = 5.1 and 16.1 Hz, 1H), 2.48-2.36 (m, 2H), 2.28-2.16 (m, 1H), 2.02 (d, J = 3.2 Hz, 3H), 2.00-1.92 (m, 1H), 1.91-1.84 (m, 1H), 1.44-1.35 (m, 1H), 1.11 (d, J = 6.7 Hz, 3H).

^{13}C NMR (100 MHz, $CDCl_3$) δ (ppm) 151.7, 147.4 (d, J = 245.2 Hz), 139.6, 138.7, 133.4, 132.5, 129.6, 124.8 (m), 123.1, 120.0, 119.8, 118.5, 118.2 (dd, J = 3.1 and 12.3 Hz), 114.5, 112.1, 31.4, 31.1, 29.5, 21.4, 20.1, 9.3 (d, J = 7.6 Hz).

Elemental analysis: calcd (%) for $C_{23}H_{18}F_3NO$ (381.40): C 72.43, H 4.76; found: C 72.28, H 5.01.

3,6-Dimethyl-2-(4,5,6-trifluoro-4'-nitro-[1,1'-biphenyl]-3-yl)-4,5,6,7-tetrahydrobenzofuran (12): Following the procedure **B** using 3,6-dimethyl-2-(2,3,4-trifluorophenyl)-4,5,6,7-tetrahydrobenzofuran (**3**) (280 mg, 1 mmol) and 1-bromo-4-nitrobenzene (303 mg, 1.5 mmol). The residue was purified by flash chromatography on silica gel (pentane–Et_2O, 65:35) to afford the desired compound **12** (0.213 g, 53%) as a dark yellow solid (mp = 100–105 ºC).

^{1}H NMR (400 MHz, $CDCl_3$**)** δ (ppm) 8.33 (d, J = 9.0 Hz, 2H), 7.72 (d, J = 8.6 Hz, 2H), 7.35 (dt, J = 2.3 and 7.4 Hz, 1H), 2.72 (dd, J = 5.4 and 16.3 Hz, 1H), 2.47-2.35 (m, 2H), 2.26-2.18 (m, 1H), 2.03 (d, J = 3.3 Hz, 3H), 2.00-1.92 (m, 1H), 1.91-1.84 (m, 1H), 1.46-1.35 (m, 1H), 1.11 (d, J = 6.7 Hz, 3H).

^{13}C NMR (100 MHz, $CDCl_3$) δ (ppm) 150.8, 146.6, 146.5 (dm, J = 264.1 Hz), 142.4 (dm, J = 264.1 Hz), 139.5, 138.5, 128.7, 123.0, 122.2, 119.1, 118.8, 117.3 (dd, J = 4.5 and 11.4 Hz), 30.4, 30.1, 28.6, 20.4, 19.0, 8.2 (d, J = 7.6 Hz).

Elemental analysis: calcd (%) for $C_{22}H_{18}F_3NO_3$ (401.38): C 65.83, H 4.52; found: C 66.03, H 4.31.

3,6-Dimethyl-2-(4,5,6-trifluoro-4'-methyl-[1,1'-biphenyl]-3-yl)-4,5,6,7-tetrahydrobenzofuran (13): Following the procedure **B** using 3,6-dimethyl-2-(2,3,4-trifluorophenyl)-4,5,6,7-tetrahydrobenzofuran (**3**) (280 mg, 1 mmol) and 4-bromotoluene (257 mg, 1.5 mmol). The residue was purified by flash chromatography on silica gel (pentane–Et_2O, 65:35) to afford the desired compound **13** (0.129 g, 35%) as yellow solid (mp = 65–69 ºC).

^{1}H NMR (400 MHz, $CDCl_3$) δ (ppm) 7.35 (d, J = 8.1 Hz, 2H), 7.24-7.21 (m, 1H), 7.19 (d, J = 7.2 Hz, 2H), 2.64 (dd, J = 5.4 and 16.3 Hz, 1H), 2.41-2.35 (m, 1H), 2.33 (s, 3H), 2.31-2.24 (m, 1H), 2.20-2.11 (m, 1H), 1.94 (d, J = 3.2 Hz, 3H), 1.92-1.85 (m, 1H), 1.83-1.77 (m, 1H), 1.37-1.28 (m, 1H), 1.03 (d, J = 6.7 Hz, 3H).

^{13}C NMR (100 MHz, $CDCl_3$) δ (ppm) 151.3, 144.9 (dm, J = 251.3 Hz), 140.2, 139.4, 138.2, 131.2, 131.0, 129.4, 128.7, 126.3 (dd, J = 3.8 and 10.9 Hz), 132.2, 119.6, 119.4, 117.4 (dd, J = 4.7 and 11.7 Hz), 31.4, 31.2, 30.9, 29.6, 21.5, 20.1, 9.3 (d, J = 7.6 Hz).

Elemental analysis: calcd (%) for $C_{23}H_{21}F_3O$ (370.42): C 74.58, H 5.71; found: C 74.82, H 5.89.

4-(1-Methyl-5-(2,3,4-trifluorophenyl)pyrrol-2-yl)benzonitrile (14): Following the procedure **B** using 1-methyl-2-(2,3,4-trifluorophenyl)pyrrole (**4**) (106 mg, 0.5 mmol) and 4-bromobenzonitrile (100 mg, 0.55 mmol). The residue was purified by flash chromatography on silica gel (pentane–Et_2O, 90:10) to afford the desired compound **14** (0.128 g, 82%) as a colorless oil.

^{1}H NMR (400 MHz, $CDCl_3$) δ (ppm) 7.71 (d, *J* = 8.3 Hz, 2H), 7.57 (d, *J* = 8.3 Hz, 2H), 7.17-7.03 (m, 2H), 6.45 (d, *J* = 3.8 Hz, 1H), 6.34 (d, *J* = 3.8 Hz, 1H), 3.55 (s, 3H).

^{13}C NMR (100 MHz, $CDCl_3$) δ (ppm) 151.0 (dd, *J* = 10.9 and 253.2 Hz), 149.0 (dd, *J* = 10.9 and 253.2 Hz), 140.4 (td, *J* = 15.2 and 251.0 Hz), 137.5, 135.4, 132.4, 130.3, 128.6, 125.4 (m), 118.9, 118.7 (dd, *J* = 3.6 and 12.3 Hz), 112.4 (dd, *J* = 4.4 and 16.9 Hz), 112.2, 110.8, 110.2, 33.9 (d, *J* = 4.2 Hz).

Elemental analysis: calcd (%) for $C_{18}H_{11}F_3N_2$ (312.30): C 69.23, H 3.55; found: C 69.51, H 3.87.

4-(5-Pentyl-3-(2,3,4-trifluorophenyl)thiophen-2-yl)benzonitrile (15): Following the procedure **B** using 2-pentyl-4-(2,3,4-trifluorophenyl)thiophene (**5**) (284 mg, 1 mmol) and 4-bromobenzonitrile (273 mg, 1.5 mmol). The residue was purified by flash chromatography on silica gel (pentane-Et_2O, 70:30) to afford the desired compound **15** (0.262 g, 68%) as a white solid (mp = 98–103 ºC).

^{1}H NMR (400 MHz, $CDCl_3$) δ (ppm) 7.53 (d, *J* = 8.3 Hz, 2H), 7.31 (d, *J* = 8.3 Hz, 2H), 6.89-6.86 (m, 2H), 6.81 (s, 1H), 2.85 (t, *J* = 7.6 Hz, 2H), 1.78-1.70 (m, 2H), 1.45-1.33 (m, 4H), 0.93 (t, *J* = 6.8 Hz, 3H).

^{13}C NMR (100 MHz, $CDCl_3$) δ (ppm) 150.8 (dd, *J* = 12.2 and 248.8 Hz), 148.8 (dd, *J* = 12.2 and 248.8 Hz), 147.1, 140.4 (td, *J* = 12.2 and 248.8 Hz), 138.9, 136.3, 132.4, 130.6, 128.7, 128.0, 124.9 (m), 121.8 (dd, *J* = 4.0 and 11.7 Hz), 118.6, 112.3 (dd, *J* = 4.2 and 16.9 Hz), 110.8, 31.3, 31.1, 30.1, 22.4, 14.0.

Elemental analysis: calcd (%) for $C_{22}H_{18}F_3NS$ (385.45): C 68.55, H 4.71; found: C 68.24, H 5.07.

4-(5-Pentyl-3-(4,5,6-trifluoro-4'-formyl-[1,1'-biphenyl]-3-yl)thiophen-2-yl)benzonitrile (16): Following the procedure **B** using 4-(5-pentyl-3-(2,3,4-trifluorophenyl)thiophen-2-yl)benzonitrile (**15**) (385 mg, 1 mmol) and 4-bromobenzaldehyde (278 mg, 1.5 mmol). The residue was purified by flash chromatography on silica gel (pentane–Et_2O, 60:40) to afford the desired compound **16** (0.296 g, 60%) as a pale yellow (mp = 104–108 ºC).

^{1}H NMR (400 MHz, $CDCl_3$) δ (ppm) 10.10 (s, 1H), 7.94 (d, *J* = 8.2 Hz, 2H), 7.58 (d, *J* = 8.3 Hz, 2H), 7.53 (d, *J* = 7.8 Hz, 2H), 7.37 (d, *J* = 8.2Hz, 2H), 7.06 (dt, *J* = 2.5 and 7.3 Hz, 1H), 6.86 (s, 1H), 2.86 (t, *J* = 7.5 Hz, 2H), 1.79-1.69 (m, 2H), 1.43-1.35 (m 4H), 0.92 (t, *J* = 7.5 Hz, 3H).

^{13}C NMR (100 MHz, $CDCl_3$) δ (ppm) 191.6, 148.3 (dd, *J* = 12.2 and 249.7 Hz), 148.1 (dd, *J* = 12.2 and 249.7 Hz), 147.3, 145.8 (dd, *J* = 12.2 and 249.7 Hz), 139.5, 138.9, 136.6, 136.0, 134.1, 132.5, 130.2, 130.0, 129.4, 128.8, 125.4, 125.1 (d, *J* = 11.6 Hz), 121.8 (dd, *J* = 4.0 and 13.2 Hz), 118.6, 111.0, 31.3, 31.2, 30.1, 22.5, 14.0.

Elemental analysis: calcd (%) for $C_{29}H_{22}F_3NOS$ (489.56): C 71.15, H 4.53; found: C 71.32, H 4.31.

Ethyl 4-(3-(2,3,4-trifluorophenyl)benzothiophen-2-yl)benzoate (17): Following the procedure **B** using 3-(2,3,4-trifluorophenyl)benzothiophene (**6**) (264 mg, 1 mmol) and 4-ethyl 4-bromobenzoate (344 mg, 1.5 mmol). The residue was purified by flash chromatography on silica gel (pentane–Et_2O, 65:35) to afford the desired compound **17** (0.301 g, 73%) as a white solid (mp = 140–145 ºC).

^{1}H NMR (400 MHz, $CDCl_3$) δ (ppm) 7.99 (d, *J* = 8.3 Hz, 2H), 7.90 (d, *J* = 7.0 Hz, 1H), 7.46 (t, *J* = 7.2 Hz, 1H), 7.42-7.37 (m, 4H), 7.07-6.97 (m, 2H), 4.39 (q, *J* = 7.2 Hz, 2H), 1.40 (t, *J* = 7.2 Hz, 3H).

^{13}C NMR (100 MHz, $CDCl_3$) δ (ppm) 166.0, 151.0 (ddd, *J* = 2.6, 10.3 and 252.0 Hz), 149.3 (ddd, *J* = 2.6, 10.3 and 252.0 Hz), 141.1, 140.5 (td, *J* = 10.3 and 252.0 Hz), 139.9, 138.9, 138.0, 130.1, 128.8, 128.8, 125.8 (m), 125.7, 125.2, 124.9, 122.9, 122.2, 120.4 (dd, *J* = 4.5 and 13.7 Hz), 112.5 (dd, *J* = 3.9 and 17.1 Hz), 61.1, 14.3.

Elemental analysis: calcd (%) for $C_{23}H_{15}F_3O_2S$ (412.43): C 66.98, H 3.67; found: C 70.22, H 3.89.

Ethyl 4-(3-(4'-cyano-4,5,6-trifluoro-[1,1'-biphenyl]-3-yl)benzothiophen-2-yl)benzoate (18): Following the procedure **B** using ethyl 4-(3-(2,3,4-trifluorophenyl)benzothiophen-2-yl)benzoate (**17**) (412 mg, 1 mmol) and 4-bromobenzonitrile (273 mg, 1.5 mmol). The residue was purified by flash chromatography on silica gel (pentane–Et_2O, 55:45) to afford the desired compound **18** (0.272 g, 53%) as a white solid (mp = 172–177 ºC).

^{1}H NMR (400 MHz, $CDCl_3$) δ (ppm) 8.01 (d, *J* = 8.3 Hz, 2H), 7.93 (d, *J* = 7.7 Hz, 1H), 7.72 (d, *J* = 8.3 Hz, 2H), 7.55-7.48 (m, 3H), 7.46-7.39 (m, 4H), 7.14 (ddd, *J* = 2.4, 6.9 and 7.7 Hz, 2H), 4.38 (q, *J* = 7.1 Hz, 2H), 1.39 (t, *J* = 7.1 Hz, 3H).

^{13}C NMR (100 MHz, $CDCl_3$) δ (ppm) 167.0, 149.2 (dd, *J* = 16.1 and 252.3 Hz), 148.4 (dd, *J* = 16.1 and 252.3 Hz), 141.6, 141.1 (td, *J* = 16.1 and 252.3 Hz), 139.7, 139.0, 138.1, 138.0, 132.5, 130.3, 130.0, 129.5, 128.9, 126.5, 125.5, 125.2, 124.8 (dd, *J* = 3.3 and 10.5 Hz), 122.8, 122.5, 120.8 (dd, *J* = 4.1 and 13.3 Hz), 118.4, 112.4, 61.3, 14.4.

Elemental analysis: calcd (%) for $C_{30}H_{18}F_3NO_2S$ (513.53): C 70.17, H 3.53; found: C 66.87, H 5.68.

2-*n*-Butyl-5-(2,4-difluorophenyl)furan (19): Following the procedure **A** using 2-*n*-butylfuran (525 µl, 3.75 mmol) and 2,4-difluorobenzenesulfonyl chloride (335 µL, 2.5 mmol). The residue was purified by flash chromatography on silica gel (pentane, 100%) to afford the desired compound **19** (0.402 g, 68%) as a colorless oil.

^{1}H NMR (400 MHz, $CDCl_3$) δ (ppm) 7.75 (dt, *J* = 6.5 and 8.8 Hz, 1H), 6.94-6.84 (m, 2H), 6.67 (t, *J* = 3.6 Hz, 1H), 6.10 (d, *J* = 3.2 Hz, 1H), 2.69 (t, *J* = 7.5 Hz, 2H), 1.68 (quint, *J* = 7.5 Hz, 2H), 1.42 (sext, *J* = 7.5 Hz, 2H), 0.96 (t, *J* = 7.5 Hz, 3H).

^{13}C NMR (100 MHz, $CDCl_3$) δ (ppm) 161.3 (dd, *J* = 13.5 and 247.7 Hz), 158.3 (dd, *J* = 13.5 and 247.7 Hz), 156.4, 145.4, 126.4 (dd, *J* = 5.0 and 9.2 Hz), 116.1 (dd, *J* = 3.7 and 11.7 Hz), 111.4 (dd, *J* = 3.0 and 21.2 Hz), 110.3 (d, *J* = 11.1 Hz), 107.2, 104.3 (t, *J* = 25.6 Hz), 30.2, 27.8, 22.3, 13.8.

Elemental analysis: calcd (%) for $C_{14}H_{14}F_2O$ (236.26): C 71.17, H 5.97; found: C 71.43, H 6.11.

2-(2,4-Difluorophenyl)benzofuran (20): Following the procedure **A** using benzofuran (413 µl, 3.75 mmol) and 2,4-difluorobenzenesulfonyl chloride (335 µL, 2.5 mmol). The residue was purified by flash chromatography on silica gel (pentane, 100%) to afford the desired compound **20** (0.368 g, 64%) as a white solid (mp = 74–78 ºC).

^{1}H NMR (400 MHz, $CDCl_3$) δ (ppm) 8.03 (dt, *J* = 6.4 and 8.8 Hz, 1H), 7.65 (d, *J* = 7.6 Hz, 1H), 7.57 (d, *J* = 8.0 Hz, 1H), 7.37 (t, *J* = 7.3 Hz, 1H), 7.30 (t, *J* = 7.2 Hz, 1H), 7.21 (d, *J* = 3.7 Hz, 1H), 7.06-6.94 (m, 2H).

^{13}C NMR (100 MHz, $CDCl_3$) δ (ppm) 162.5 (dd, *J* = 13.7 and 251.5 Hz), 159.6 (dd, *J* = 13.7 and 251.5 Hz), 154.1, 148.9, 129.2, 127.9 (dd, *J* = 4.4 and 9.6 Hz), 124.7, 123.0, 121.3, 115.3 (dd, *J* = 3.9 and 11.9 Hz), 111.7 (dd, *J* = 3.4 and 21.7 Hz), 110.9, 105.9 (d, *J* = 12.5 Hz), 104.5 (t, J = 25.7 Hz).

Elemental analysis: calcd (%) for $C_{14}H_8F_2O$ (230.21): C 73.04, H 3.50; found: C 73.29, H 3.59.

2-(2,4-Difluorophenyl)-3,6-dimethyl-4,5,6,7-tetrahydrobenzofuran (21): Following the procedure **A** using menthofuran (387 µl, 3.75 mmol) and 2,4-difluorobenzenesulfonyl chloride (335 µL, 2.5 mmol). The residue was purified by flash chromatography on silica gel (pentane, 100%) to afford the desired compound **21** (0.538 g, 82%) as a colorless oil.

^{1}H NMR (400 MHz, $CDCl_3$) δ (ppm) 7.48 (dt, J = 6.6 and 8.5 Hz, 1H), 6.97-6.86 (m, 2H), 2.75 (dd, J = 5.2 and 16.2 Hz, 1H), 2.50-2.35 (m, 2H), 2.30-2.21 (m, 1H), 2.01 (d, J = 2.8 Hz, 3H), 2.00-1.94 (m, 1H), 1.93-1.85 (m, 1H), 1.47-1.36 (m, 1H), 1.13 (d, J = 6.7 Hz, 3H).

^{13}C NMR (100 MHz, $CDCl_3$) δ (ppm) 162.1 (dd, J = 12.2 and 246.9 Hz), 159.0 (dd, J = 12.2 and 246.9 Hz), 150.7, 141.2, 130.6 (dd, J = 5.0 and 9.5 Hz), 119.3, 118.4, 116.63 (dd, J = 3.8 and 14.7 Hz), 111.3 (dd, J = 3.3 and 21.4 Hz), 104.3 (t, J = 25.7 Hz), 31.5, 31.4, 29.7, 21.5, 20.2, 9.09 (d, J = 6.5 Hz).

Elemental analysis: calcd (%) for $C_{16}H_{16}F_2O$ (262.29): C 73.27, H 6.15; found: C 73.61, H 6.34.

2-(2,4-Difluorophenyl)-5-(4-methoxyphenyl)-1-methylpyrrole (22):

Following the procedure **A** using 2-(4-methoxyphenyl)-1-methylpyrrole (387 µl, 3.75 mmol) and 2,4-difluorobenzenesulfonyl chloride (335 µL, 2.5 mmol) The residue was purified by flash chromatography on silica gel (pentane, 100%) to afford the desired compound **21** (0.650 g, 87%) as a white solid (mp = 136–140 ºC).

^{1}H NMR (400 MHz, $CDCl_3$) δ (ppm) 7.41 (d, J = 8.5 Hz, 2H), 7.39-7.34 (m, 1H), 6.98 (d, J = 8.5 Hz, 2H), 6.96-6.90 (m, 2H), 6.27 (s, 2H), 3.87 (s, 3H), 3.47 (s, 3H).

^{13}C NMR (100 MHz, $CDCl_3$) δ (ppm) 162.5, 159.9 (d, J = 253.3 Hz), 158.9, 136.5, 132.9 (dd, J = 4.0 and 9.5 Hz), 130.1, 128.6, 126.0, 117.9 (dd, J = 3.7 and 15.9 Hz), 113.9, 111.5 (dd, J = 3.0 and 20.8 Hz), 109.8, 108.0, 104.2 (t, J = 24.5 Hz), 55.4, 33.4.

Elemental analysis: calcd (%) for $C_{18}H_{15}F_2NO$ (299.32): C 72.23, H 5.05; found: C 72.53, H 5.29.

2-(3,4-Difluorophenyl)-3,6-dimethyl-4,5,6,7-tetrahydrobenzofuran (23):

Following the procedure **A** using menthofuran (387 µl, 3.75 mmol) and 3,4-difluorobenzenesulfonyl chloride (336 µL, 2.5 mmol). The residue was purified by flash chromatography on silica gel (pentane, 100%) to afford the desired compound **23** (0.537 g, 82%) as a brown oil.

^{1}H NMR (400 MHz, $CDCl_3$) δ (ppm) 7.38 (ddd, J = 2.2, 7.6 and 12.1 Hz, 1H), 7.31-7.26 (m, 1H), 7.15 (td, J = 8.5 and 10.2 Hz, 1H), 2.71 (dd, J = 5.2 and 16.4 Hz, 1H), 2.46-2.30 (m, 2H), 2.27-2.16 (m, 1H), 2.13 (s, 3H), 1.99-1.90 (m, 1H), 1.89-1.81 (m, 1H), 1.44-1.34 (m, 1H), 1.10 (d, J = 6.7 Hz, 3H).

^{13}C NMR (100 MHz, $CDCl_3$) δ (ppm) 150.4 (dd, J = 8.5 and 246.7 Hz), 150.0, 148.6 (dd, J = 8.5 and 246.7 Hz), 144.8, 129.6 (dd, J = 3.7 and 5.1 Hz), 120.6 (m), 120.0, 117.3 (d, J = 17.8 Hz), 116.7, 113.6 (d, J = 19.0 Hz), 31.6, 31.2, 29.6, 21.4, 20.0, 9.8.

Elemental analysis: calcd (%) for $C_{16}H_{16}F_2O$ (262.29): C 73.27, H 6.15; found: C 73.46, H 6.37.

2-(3,4-Difluorophenyl)-5-(4-methoxyphenyl)-1-methylpyrrole (24):

Following the procedure **A** using 2-(4-methoxyphenyl)-1-methylpyrrole (387 µl, 3.75 mmol) and 3,4-difluorobenzenesulfonyl chloride (336 µL, 2.5 mmol). The residue was purified by flash chromatography on silica gel (pentane, 100%) to afford the desired compound **24** (0.591 g, 79%) as a white solid (mp = 122–126 ºC).

^{1}H NMR (400 MHz, $CDCl_3$) δ (ppm) 7.40 (d, J = 8.5 Hz, 2H), 7.33-7.14 (m, 3H), 6.99 (d, J = 8.5 Hz, 2H), 6.30 (d, J = 3.5 Hz, 1H), 6.26 (d, J = 3.5 Hz, 1H), 3.87 (s, 3H), 3.57 (s, 3H).

^{13}C NMR (100 MHz, $CDCl_3$) δ (ppm) 158.9, 150.2 (dd, J = 12.5 and 248.5 Hz), 149.4 (dd, J = 12.5 and 248.5 Hz), 137.3, 134.1, 130.8 (dd, J = 3.8 and 4.9 Hz), 130.2, 127.8, 125.9, 124.6 (dd, J = 3.5 and 5.5 Hz), 117.3 (d, J = 17.5 Hz), 114.0, 109.2, 108.2, 55.4, 34.1.

Elemental analysis: calcd (%) for $C_{18}H_{15}F_2NO$ (299.32): C 72.23, H 5.05; found: C 72.46, H 5.32.

5'-(3,6-Dimethyl-4,5,6,7-tetrahydrobenzofuran-2-yl)-2',3'-difluoro-[1,1'-biphenyl]-4-carbaldehyde (25):

Following the procedure **B** using 2-(3,4-difluorophenyl)-3,6-dimethyl-4,5,6,7-tetrahydrobenzofuran (**23**) (262 mg, 1 mmol) and 4-bromobenzaldehyde (278 mg, 1.5 mmol). The residue was purified by flash chromatography on silica gel (pentane–Et_2O, 60:40) to afford the desired compound **25** (0.194 g, 53%) as a brown oil.

^{1}H NMR (400 MHz, $CDCl_3$) δ (ppm) 10.07 (s, 1H), 7.98 (d, *J* = 8.2 Hz, 2H), 7.75 (d, *J* = 8.2 Hz, 2H), 7.44-7.37 (m, 2H), 2.72 (dd, *J* = 5.4 and 16.5 Hz, 1H), 2.48-2.31 (m, 2H), 2.28-2.18 (m, 1H), 2.16 (s, 3H), 2.00-1.91 (m, 1H), 1.91-1.82 (m, 1H), 1.45-1.34 (m, 1H), 1.11 (d, *J* = 6.7 Hz, 3H).

^{13}C NMR (100 MHz, $CDCl_3$) δ (ppm) 191.7, 151.0 (dd, *J* = 12.9 and 247.9 Hz), 150.3, 146.0 (dd, *J* = 12.9 and 247.9 Hz), 144.4, 140.9, 135.8, 129.9, 129.7, 129.61 (d, *J* = 3.0 Hz), 129.3 (dd, *J* = 5.0 and 6.0 Hz), 121.1, 120.1, 117.3, 113.1 (d, *J* = 19.7 Hz), 31.3, 31.1, 29.6, 21.4, 19.9, 9.8.

Elemental analysis: calcd (%) for $C_{23}H_{20}F_2O_2$ (366.41): C 75.39, H 5.50; found: C 75.17, H 5.89.

Ethyl 3'-(3,6-dimethyl-4,5,6,7-tetrahydrobenzofuran-2-yl)-2',6'-difluoro-[1,1'-biphenyl]-4-carboxylate (26):

Following the procedure **B** using 2-(2,4-difluorophenyl)-3,6-dimethyl-4,5,6,7-tetrahydrobenzofuran (**21**) (262 mg, 1 mmol) and ethyl 4-bromobenzoate (344 mg, 1.5 mmol). The residue was purified by flash chromatography on silica gel (pentane–Et_2O, 65:35) to afford the desired compound **26** (0.279 g, 68%) as a colorless oil.

^{1}H NMR (400 MHz, $CDCl_3$) δ (ppm) 8.15 (d, *J* = 8.2 Hz, 2H), 7.58 (d, *J* = 8.2 Hz, 2H), 7.48 (ddd, *J* = 6.4, 8.0 and 8.7 Hz, 1H), 7.05 (t, *J* = 8.9 Hz, 1H), 4.41 (q, *J* = 7.1 Hz, 2H), 2.73 (dd, *J* = 5.1 and 16.3 Hz, 1H), 2.49-2.32 (m, 2H), 2.28-2.20 (m, 1H), 1.99 (d, *J* = 2.5 Hz, 3H), 1.98-1.92 (m, 1H), 1.91-1.83 (m, 1H), 1.42 (t, *J* = 7.2 Hz, 3H), 1.40-1.34 (m, 1H), 1.11 (d, *J* = 6.7 Hz, 3H).

^{13}C NMR (100 MHz, $CDCl_3$) δ (ppm) 166.5, 191.1 (dd, *J* = 9.4 and 248.8 Hz), 155.9 (dd, *J* = 9.4 and 248.8 Hz), 151.1, 141.2, 130.6, 130.4, 129.8 (m), 129.6, 129.0, 119.6, 118.9, 118.2 (t, *J* = 19.3 Hz), 117.2 (dd, *J* = 3.0 and 16.7 Hz), 111.9 (dd, *J* = 3.0 and 23.1 Hz), 61.3, 31.6, 31.4, 29.8, 21.7, 20.3, 14.5, 9.4 (d, *J* = 6.4 Hz).

Elemental analysis: calcd (%) for $C_{25}H_{24}F_2O_3$ (410.46): C 73.16, H 5.89; found: C 73.37, H 6.13.

3'-(5-Butylfuran-2-yl)-2',6'-difluoro-[1,1'-biphenyl]-4-carbonitrile (27):

Following the procedure **B** using 2-butyl-5-(2,4-difluorophenyl)furan (**19**) (236 mg, 1 mmol) and 4-bromobenzonitrile (273 mg, 1.5 mmol). The residue was purified by flash chromatography on silica gel (pentane–Et_2O, 75:25) to afford the desired compound **27** (0.098 g, 29%) as a yellow oil.

^{1}H NMR (400 MHz, $CDCl_3$) δ (ppm) 7.82 (dt, *J* = 6.1 and 8.7 Hz, 1H), 7.76 (d, *J* = 8.2 Hz, 2H), 7.61 (d, *J* = 8.2 Hz, 2H), 7.06 (dt, *J* = 1.5 and 8.9 Hz, 1H), 6.69 (t, *J* = 3.8 Hz, 1H), 6.12 (d, *J* = 3.3 Hz, 1H), 2.7 (t, *J* = 7.5 Hz, 2H), 1.69 (quint, *J* = 7.5 Hz, 2H), 1.42 (sext, *J* = 7.5 Hz, 2H), 0.96 (t, *J* = 7.5 Hz, 3H).

^{13}C NMR (100 MHz, $CDCl_3$) δ (ppm) 158.0 (dd, *J* = 6.8 and 250.9 Hz), 156.8, 154.9 (dd, *J* = 6.8 and 250.9 Hz), 145.0, 134.2, 131.2, 126.1 (dd, *J* = 5.6 and 9.3 Hz), 118.6, 117.0 (m), 112.2, 112.0 (dd *J* = 4.0 and 23.1 Hz), 110.9, 110.8, 107.4, 105.2 (t, *J* = 25.9 Hz), 30.2, 27.8, 22.3, 13.8.

Elemental analysis: calcd (%) for $C_{21}H_{17}F_2NO$ (337.37): C 74.76, H 5.08; found: C 74.95, H 5.31.

3-(3-(Benzofuran-2-yl)-2,6-difluorophenyl)quinoline (28):

Following the procedure **B** using **2**-(2,4-difluorophenyl)benzofuran (**20**) (230 mg, 1 mmol) and 3-bromoquinoline (312 mg, 1.5 mmol). The residue was purified by flash chromatography on silica gel (pentane–Et_2O, 75:25) to afford the desired compound **28** (0.200 g, 56%) as a pale yellow solid (mp = 130–135 ºC).

^{1}H NMR (400 MHz, $CDCl_3$) δ (ppm) 9.01 (s, 1H), 8.35 (s, 1H), 8.2 (d, *J* = 8.3 Hz, 1H), 8.10 (dt, *J* = 6.5 and 8.7 Hz, 1H), 7.91 (d, *J* = 8.0 Hz, 1H), 7.80 (t, *J* = 6.9 Hz, 1H), 7.65-7.59 (m, 2H), 7.55 (d, *J* = 8.0 Hz, 1H), 7.33 (t, *J* = 7.8 Hz, 1H), 7.28-7.17 (m, 3H).

^{13}C NMR (100 MHz, $CDCl_3$) δ (ppm) 159.7 (dd, *J* = 6.1 and 252.0 Hz), 156.8 (dd, *J* = 6.1 and 252.0 Hz), 154.2, 151.2, 148.8, 147.6, 137.7, 130.3, 129.4, 129.2, 128.1, 127.6, 127.4 (dd, *J* = 5.2 and 10.4 Hz), 127.1, 125.0, 123.2, 122.4, 121.5, 116.1 (dd, *J* = 5.1 and 11.4 Hz), 115.9 (t, *J* = 19.2 Hz), 112.3 (dd, *J* = 4.4 and 22.4 Hz), 111.1, 106.5 (d, *J* = 12.7 Hz).

Elemental analysis: calcd (%) for $C_{23}H_{13}F_2NO$ (357.36): C 77.30, H 3.67; found: C 77.91, H 4.02.

3-(2,6-Difluoro-3-(5-(4-methoxyphenyl)-1-methylpyrrol-2-yl)phenyl)quinoline (29):

Following the procedure **B** using 2-(3,4-difluorophenyl)-5-(4-methoxyphenyl)-1-methylpyrrole (**24**) (299 mg, 1 mmol) and 3-bromoquinoline (312 mg, 1.5 mmol). The residue was purified by flash chromatography on silica gel (pentane–Et_2O, 65:45) to afford the desired compound **29** (0.218 g, 51%) as a white solid (mp = 124–128 ºC).

^{1}H NMR (400 MHz, $CDCl_3$) δ (ppm) 9.07 (s, 1H), 8.36 (s, 1H), 8.20 (d, *J* = 8.5 Hz, 1H), 7.90 (d, *J* = 8.4 Hz, 1H), 7.80 (t, *J* = 7.8 Hz, 1H), 7.62 (dd, *J* = 7.0 and 8.1 Hz, 1H), 7.47 (dt, *J* = 6.4 and 8.6 Hz, 1H), 7.40 (d, *J* = 8.8 Hz, 2H), 7.15 (t, *J* = 8.63 Hz, 1H), 6.97 (d, *J* = 8.8 Hz, 2H), 6.35 (d, *J* = 3.7 Hz, 1H), 6.29 (d, *J* = 3.7 Hz, 1H), 3.85 (s, 3H), 3.52 (s, 3H).

^{13}C NMR (100 MHz, $CDCl_3$) δ (ppm) 159.6 (dd, *J* = 9.1 and 247.6 Hz), 158.9, 157.1 (dd, *J* = 9.1 and 247.6 Hz), 151.2, 147.2, 137.8, 136.9, 132.2 (dd, *J* = 5.9 and 8.8 Hz), 130.3, 130.2, 129.2, 128.4, 128.1, 127.7, 127.2, 125.9, 122.8, 118.7 (dd, *J* = 3.4 and 7.1 Hz), 115.4 (t, *J* = 19.0 Hz), 113.9, 112.0 (dd, *J* = 3.5 and 22.6 Hz), 110.2, 108.2, 55.4, 33.5 (d, *J* = 4.3 Hz).

Elemental analysis: calcd (%) for $C_{27}H_{20}F_2N_2O$ (426.47): C 76.04, H 4.73; found: C 76.27, H 4.52.

2-(2-Chloro-4-fluorophenyl)-3,6-dimethyl-4,5,6,7-tetrahydrobenzofuran (30):

Following the procedure **A** using menthofuran (387 µl, 3.75 mmol) and 2-chloro-4-fluorobenzenesulfonyl chloride (365 µL, 2.5 mmol). The residue was purified by flash chromatography on silica gel (pentane–Et_2O, 90:10) to afford the desired compound **30** (0.515 g, 74%) as a brown oil. ^{1}H NMR (400 MHz, $CDCl_3$) δ (ppm) 7.38 (dd, *J* = 6.1 and 8.7 Hz, 1H), 7.2 (dd, *J* = 2.5 and 8.6 Hz, 1H), 7.01 (dt, *J* = 2.9 and 9.1 Hz, 1H), 2.73 (dd, *J* = 5.3 and 16.3 Hz, 1H), 2.48-2.35 (m, 2H), 2.28-2.20 (m, 1H), 2.03-1.95 (m, 1H), 1.94 (s, 3H), 1.91-1.84 (m, 1H), 1.47-1.36 (m, 1H), 1.11 (d, *J* = 6.7 Hz, 3H). ^{13}C NMR (100 MHz, $CDCl_3$) δ (ppm) 161.9 (d, *J* = 253.3 Hz), 150.4, 143.8, 134.3 (d, *J* = 10.2 Hz), 132.7 (d, *J* = 8.7 Hz), 127.5 (d, *J* = 4.1 Hz), 118.8, 118.3, 117.3 (d, *J* = 24.5 Hz), 113.8 (d, *J* = 20.4 Hz), 31.4, 31.3, 29.7, 21.6, 20.2, 9.4.

Elemental analysis: calcd (%) for $C_{16}H_{16}ClFO$ (278.75): C 68.94, H 5.79; found: C 69.16, H 5.57.

Ethyl 2'-chloro-3'-(3,6-dimethyl-4,5,6,7-tetrahydrobenzofuran-2-yl)-6'-fluoro-[1,1'-biphenyl]-4-carboxylate (31):

Following the procedure **B** using 2-(2-chloro-4-fluorophenyl)-3,6-dimethyl-4,5,6,7-tetrahydrobenzofuran (**30**) (279 mg, 1 mmol) and 4-ethyl 4-bromobenzoate (344 mg, 1.5 mmol). The residue was purified by flash chromatography on silica gel (pentane–Et_2O, 65:35) to afford the desired compound **31** (0.196 g, 46%) as colorless oil. ^{1}H NMR (400 MHz, $CDCl_3$) δ (ppm) 8.15 (d, *J* = 8.2 Hz, 2H), 7.64 (dd, *J* = 8.3 and 13.9 Hz, 1H), 7.46 (d, *J* = 8.2 Hz, 2H), 7.41 (dd, *J* = 6.2 and 9.0 Hz, 1H), 7.13 (t, *J* = 8.6 Hz, 1H), 4.41 (q, *J* = 7.1 Hz, 2H), 2.72 (dd, *J* = 5.1 and 16.7 Hz, 1H), 2.48-2.33 (m, 2H), 2.28-2.18 (m, 1H), 1.94 (s, 3H), 1.91-1.84 (m, 1H), 1.42 (t, *J* = 7.13 Hz, 3H), 1.11 (d, *J* = 6.7 Hz, 3H). ^{13}C NMR (100 MHz, $CDCl_3$) δ (ppm) 166.3, 159.4 (d, *J* = 251.3 Hz), 150.5, 144.2, 137.7, 133.6, 132.0 (d, *J* = 9.5 Hz), 130.3, 130.1, 129.4, 128.9, 128.2 (d, *J* = 3.2

Hz), 127.1 (d, J = 26.6 Hz), 118.5 (d, J = 31.6 Hz), 114.0 (d, J = 23.9 Hz), 61.1, 31.5, 31.3, 29.7, 21.6, 20.2, 14.4, 9.5. Elemental analysis: calcd (%) for $C_{25}H_{24}ClFO_3$ (426.91): C 70.34, H 5.67; found: C 70.73, H 5.89.

4-(2-Chloro-4-fluorophenyl)-2-pentylthiophene (32):

Following the procedure **A** using 2-pentylthiophene (605 µl, 3.75 mmol) and 2-chloro-4-fluorobenzenesulfonyl chloride (365 µL, 2.5 mmol). The residue was purified by flash chromatography on silica gel (pentane–Et_2O, 85:15) to afford the desired compound **32** (0.551 g, 78%) as a colorless oil. ^1H NMR (400 MHz, $CDCl_3$) δ (ppm) 7.38 (dd, J = 6.2 and 8.6 Hz, 1H), 7.22-7.17 (m, 2H), 7.00 (dt, J = 2.6 and 8.5 Hz, 1H), 6.94 (s, 1H), 2.85 (t, J = 7.7 Hz, 2H), 1.78-1.69 (m, 2H), 1.44-1.35 (m, 4H), 0.94 (t, J = 7.7 Hz, 3H). ^{13}C NMR (100 MHz, $CDCl_3$) δ (ppm) 161.5 (d, J = 249.5 Hz), 145.5, 137.9, 133.1 (d, J = 10.3 Hz), 132.1 (d, J = 3.9 Hz), 131.9 (d, J = 8.9 Hz), 125.7, 121.6, 117.4 (d, J = 24.8 Hz), 114.0 (d, J = 20.6 Hz), 31.3, 30.1, 22.5, 14.1.

Elemental analysis: calcd (%) for $C_{15}H_{16}ClFS$ (282.80): C 63.71, H 5.70; found: C 63.95, H 6.04.

3-(3-(2-Chloro-4-fluorophenyl)-5-pentylthiophen-2-yl)quinoline (33):

Following the procedure **B** using **4**-(2-chloro-4-fluorophenyl)-2-pentylthiophene (**32**) (283 mg, 1 mmol) and 3-bromoquinoline (312 mg, 1.5 mmol). The residue was purified by flash chromatography on silica gel (pentane–Et_2O, 60:40) to afford the desired compound **33** (0.299 g, 73%) as a yellow oil.

^1H NMR (400 MHz, $CDCl_3$) δ (ppm) 8.66 (s, 1H), 8.03 (d, J = 9.0 Hz, 1H), 7.96 (s, 1H), 7.72-7.62 (m, 2H), 7.50 (dd, J = 6.9 and 8.0 Hz, 1H), 7.20-7.16 (m, 2H), 6.90 (dt, J = 2.6 and 8.1 Hz, 1H), 6.83 (s, 1H) 2.88 (t, J = 7.4 Hz, 2H), 1.77 (quint, J = 7.4 Hz, 2H), 1.48-1.36 (m, 4H), 0.94 (t, J = 7.4 Hz, 3H).

^{13}C NMR (100 MHz, $CDCl_3$) δ (ppm) 162.0 (d, J = 247.8 Hz), 150.0, 146.6, 145.8, 135.3, 134.4 (d, J = 10.4 Hz), 134.2, 132.9 (d, J = 8.5 Hz), 131.8 (d, J = 3.6 Hz), 129.6, 129.1, 128.2, 127.9 127.8, 127.7, 127.1, 117.4 (d, J = 26.0 Hz), 144.4 (d, J = 21.4 Hz), 31.4, 31.2, 29.8, 22.5, 14.0.

Elemental analysis: calcd (%) for $C_{24}H_{21}ClFNS$ (409.95): C 70.32, H 5.16; found: C 70.53, H 4.87.

2-*n*-Butyl-5-(perfluorophenyl)furan (34):

Following the procedure **A** using 2-*n*-butylfuran (210 µl, 1.5 mmol) and pentafluorobenzenesulfonyl chloride (147 µL, 1 mmol). The residue was purified by flash chromatography on silica gel (pentane–Et_2O, 98:2) to afford the desired compound **34** (0.238 g, 82%) as a brown oil.

^1H NMR (400 MHz, $CDCl_3$) δ (ppm) 6.78 (td, J = 2.0 and 3.3 Hz, 1H), 6.17 (d, J = 3.3 Hz, 1H), 2.72 (t, J = 7.6 Hz, 2H), 1.7 (quint., J = 7.6 Hz, 2H), 1.43 (sext., J = 7.6 Hz, 2H), 0.97 (t, J = 7.6 Hz, 3H).

^{13}C NMR (100 MHz, $CDCl_3$) δ (ppm) 158.7, 143.1 (dm, J = 255.7 Hz), 139.5 (dm, J = 255.7 Hz), 139.3 (m), 139.1 (m), 138.1 (dm, J = 255.7 Hz), 114.5 (td, J = 6.1 and 1.6 Hz), 107.0, 30.0, 27.8, 22.2, 13.7.

Elemental analysis: calcd (%) for $C_{14}H_{11}F_5O$ (290.23): C 57.94, H 3.82; found: C 58.18, H 3.61.

2-(Perfluorophenyl)benzofuran (35):

Following the procedure **A** using benzofuran (165 µl, 1.5 mmol) and pentafluorobenzenesulfonyl chloride (147 µL, 1 mmol). The residue was purified by flash chromatography on silica gel (pentane–Et_2O, 98:2) to afford the desired compound **35** (0.225 g, 79%) as a brown oil.

^1H NMR (400 MHz, $CDCl_3$) δ (ppm) 7.66 (d, J = 7.5 Hz, 1H), 7.58 (d, J = 7.8 Hz, 1H), 7.39 (dt, J = 1.3 and 7.5 Hz, 1H), 7.30 (m, 1H), 7.24 (1H, s).

This is a known compound and the spectral data are identical to those reported in literature [4].

2-*n*-Pentyl-4-(perfluorophenyl)thiophene (36):

Following the procedure **A** using 2-*n*-pentylthiophene (242 µl, 3.75 mmol) and pentafluorobenzenesulfonyl chloride (147 µL, 1 mmol) The residue was purified by flash chromatography on silica gel (pentane–Et_2O, 98:2) to afford the desired compound **36** (0.234 g, 73%) as a brown oil.

^1H NMR (400 MHz, $CDCl_3$) δ (ppm) 7.42 (s, 1H), 7.02 (s, 1H), 2.86 (t, *J* = 7.5 Hz, 2H), 1.72 (quint., *J* = 7.5 Hz, 2H), 1.43-1.32 (m, 4H), 0.92 (t, *J* = 7.5 Hz, 2H).

^{13}C NMR (100 MHz, $CDCl_3$) δ (ppm) 146.3, 144.2 (dm, *J* = 243.6 Hz), 139.7 (dm, *J* = 243.6 Hz), 137.8, 127.1, 125.0, 124.8 (t, *J* = 3.5 Hz), 124.4, 31.3, 31.2, 29.9, 22.3, 14.0.

Elemental analysis: calcd (%) for $C_{15}H_{13}F_5S$ (320.32): C 56.25, H 4.09; found: C 56.35, H 3.87.

References

[1] Cantat, T.; Génin, E.; Giroud, C.; Meyer, G.; Jutand, A. *J. Organomet. Chem.* **2003**, *687*, 365-376.

[2] Hfaiedh, A.; Yuan, K.; Ben Ammar, H.; Ben Hassine, B.; Soulé, J.-F.; Doucet, H. *ChemSusChem* **2015**, *8*, 1794-1804.

[3] Yan, T.; Zhao, L.; He, M.; Soulé, J.-F.; Bruneau, C.; Doucet, H. *Adv. Synth. Catal.* **2014**, *356*, 1586-1596.

[4] He, C.-Y.; Fan, S.; Zhang, X. *J. Am. Chem. Soc.* **2010**, *132*, 12850-12852.
